# A systematic review on descending serotonergic projections and modulation of spinal nociception in chronic neuropathic pain and after spinal cord stimulation

**DOI:** 10.1177/17448069211043965

**Published:** 2021-10-18

**Authors:** Lonne Heijmans, Martijn R Mons, Elbert A Joosten

**Affiliations:** 1 Department of Anesthesiology and Pain Management, Maastricht University Medical Centre, the Netherlands; 2 Department of Translational Neuroscience, School of Mental Health and Neuroscience, Maastricht University, the Netherlands

**Keywords:** Serotonin, 5-HT, dorsal horn, nociception, neuropathic pain, spinal cord stimulation

## Abstract

Chronic neuropathic pain is a debilitating ordeal for patients worldwide and pharmacological treatment efficacy is still limited. As many pharmacological interventions for neuropathic pain often fail, insights into the underlying mechanism and role of identified receptors is of utmost importance. An important target for improving treatment of neuropathic pain is the descending serotonergic system as these projections modulate nociceptive signaling in the dorsal horn. Also with use of last resort treatments like spinal cord stimulation (SCS), the descending serotonergic projections are known to be involved in the pain relieving effect. This systematic review summarizes the involvement of the serotonergic system on nociceptive modulation in the healthy adult rodent and the chronic neuropathic rodent and summarizes all available literature on the serotonergic system in the SCS-treated neuropathic rodent. Medline, Embase and Pubmed databases were used in the search for articles. Descending serotonergic modulation of nociceptive signaling in spinal dorsal horn in normal adult rat is mainly inhibitory and mediated by 5-HT1a, 5-HT1b, 5-HT2c, 5-HT3 and 5-HT4 receptors. Upon injury and in the neuropathic rat, this descending serotonergic modulation becomes facilitatory via activation of the 5-HT2a, 5-HT2b and 5-HT3 receptors. Analgesia due to neuromodulatory intervention like SCS restores the inhibitory function of the descending serotonergic system and involves 5-HT2, 5-HT3 and 5-HT4 receptors. The results of this systematic review provide insights and suggestions for further pharmacological and or neuromodulatory treatment of neuropathic pain based on targeting selected serotonergic receptors related to descending modulation of nociceptive signaling in spinal dorsal horn. With the novel developed SCS paradigms, the descending serotonergic system will be an important target for mechanism-based stimulation induced analgesia.

## Introduction

**Figure 1.**
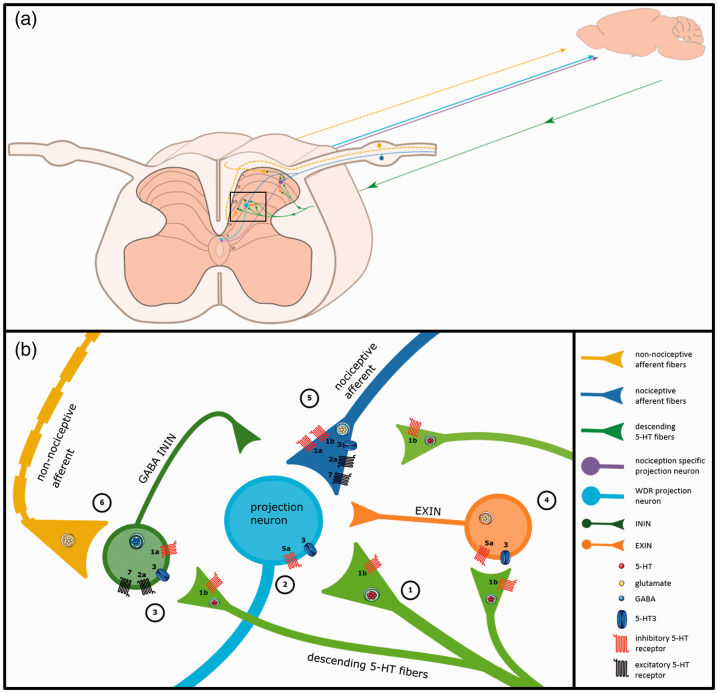
Descending serotonergic fibers and 5-HT receptors in the dorsal horn nociceptive network of the adult rat. Dorsal horn nociceptive network: Nociceptive afferent fibers (thinly myelinated Aδ-fiber, unmyelinated C-fiber) terminate in the superficial layers (lamina I-II) of the DH where they either synapse on interneurons (lamina I-III) or NK1 receptor expressing projection neurons (lamina I).^5^ Neurotransmitters utilized by inhibitory interneurons (ININs) are γ-aminobutyric acid (GABA), glycine, or both. Excitatory interneurons (EXIN) utilize glutamate.^6^ The interneurons synapse on projection neurons either in lamina I (nociceptive specific) or in lamina III-V (wide dynamic range (WDR) neurons). WDR neurons have dendrites extending to the superficial lamina and thus synapses form nociceptive fibers, non-nociceptive fibers and interneurons.^7^ Non-noxious stimuli are transmitted by touch-responsive, myelinated Aβ fibers that terminate within lamina II-V and synapse onto the WDR and interneurons.^5^ Descending serotonergic neurons terminate most abundantly in the superficial laminae (I/II) but they also innervate deeper laminae (IV-VI).^4,8,9^ Panel (b) depicts the enlarged inset of (a) and contains numbered pathways of 5-HT mediated nociceptive modulation: 1) Autoreceptor pathway; direct modulation of serotonin release through 5-HT1b autoreceptors on descending serotonergic terminals.^10^ 2) Projection neuron pathway; direct modulation through postsynaptic 5-HT3 and 5-HT5a expression on spinal projection neurons.^11,12^ 3) GABA ININ pathway; indirect modulation of projection neurons through 5-HT1a, 5-HT2a, 5-HT3 and 5-HT7 expressed on GABAergic ININs.^13–19^ 4) EXIN pathway; indirect modulation of projection neuron through 5-HT3 and 5-HT5a expression on EXINs.^11,12^ 5) Nociceptive afferent pathway; direct modulation of neurotransmitter release through expression of 5-HT1a, 5-HT1b, 5-HT2a, 5-HT3 and 5-HT7 on nociceptive afferent terminals.^10,12,14,17,19–24^ 6) Non-nociceptive afferents pathway; modulation via activation of GABAergic ININs by non-nociceptive afferents (Aβ fibers) according to the principle of the Gate-Control Theory.^25^

Chronic neuropathic pain is an important worldwide problem that negatively impacts the quality of life of patients and imposes great socioeconomic costs.^1^ Neuropathic pain is a direct consequence of damage to the somatosensory nervous system, either through lesion or disease.^2^ In neuropathic pain, the processing of nociceptive information from not only the periphery to the spinal dorsal horn (DH) is completely derailed, but central processes including descending modulatory control from brainstem areas that innervate the DH are changed as well.^1,3^ Spinal cord stimulation (SCS) as a treatment for chronic neuropathic pain might be able to alter the derailed processing of nociceptive information and the descending modulation in the DH, thereby providing analgesia. As the main neurotransmitter involved in descending modulation of spinal nociceptive neurotransmission is serotonin, the present review focuses on serotonergic descending projections. Virtually all serotonergic innervation of the spinal cord originates from supraspinal sources. Whereas the dorsal raphe nucleus (DRN) provides mainly ascending projections to other brain structures, the nucleus raphe magnus (NRM) provides serotonergic input to the spinal DH.^4^ Local 5-HT receptors mediate the net modulatory effect of 5-HT in the spinal DH (see Figure 1 and Table 1 for 5-HT receptor expression in spinal DH). Based on the receptors involved, the net effect of serotonergic descending modulation might be either inhibitory or facilitatory.^1,3,4,26^

**Table 1.** 5-HT receptor expression on different cell types within the dorsal horn of the spinal cord.

	Nociceptive afferent	Projection neuron	Inhibitory interneuron (GABAergic)	Excitatory interneuron	Descending serotonergic terminal
Inhibitory receptors					
5-HT1a	**✓**	?	**✓**	?	**–**
5-HT1b	**✓**	?	?	?	**✓**
5-HT1d	?	?	?	?	?
5-HT5a	–	**✓**	?	**✓**	**–**
Excitatory receptors					
5-HT2a	**✓**	?	**✓**	?	?
5-HT2b	?	?	?	?	?
5-HT2c	?	?	?	?	?
5-HT3	**✓**	**✓**	**✓**	**✓**	**–**
5-HT4	?	?	?	?	?
5-HT6	?	?	?	?	?
5-HT7	**✓**	?	**✓**	?	?

**✓**: receptor present on cell type; –: receptor not present on cell type; ?: unknown.

The main goal of this systematic review is to understand the role of descending serotonergic projection in chronic neuropathic pain (Descending serotonergic projections and spinal nociception in chronic neuropathic rodents section) and how SCS-induced pain relief in neuropathic pain acts via the modulation of these descending brainstem-spinal cord projections (Descending serotonergic projections and spinal nociception: Spinal cord stimulation in chronic neuropathic rodents section). The vast majority of literature is based on preclinical rodent studies. This review starts with summarizing the present literature on descending serotonergic brainstem-spinal cord projections in the normal (Descending serotonergic projections and spinal nociception in adult rodent section) and in the neuropathic rat (Descending serotonergic projections and spinal nociception in chronic neuropathic rodents section). The Descending serotonergic projections and spinal nociception: Spinal cord stimulation in chronic neuropathic rodents section summarizes literature on SCS in modulation of descending serotonergic brainstem-spinal cord projections and pain relief. In the Summary and discussion section, results are summarized and discussed, limitations and future perspectives are presented.

## Methods

A systematic literature search was conducted using Pubmed, Medline and Embase search engines. Search terms and strategy for each database are included in Appendix 1. Literature searches were performed until May 15, 2020 by one reviewer (LH). No date limits were applied to the search but selected articles were restricted to the English language. Only articles on the serotonergic system relating to either healthy nociception, peripheral chronic pain models and/or neurostimulation were included. Only preclinical studies on rodents were included. Articles included in the review must be original studies, reviews resulting from the search were screened for the inclusion of additional articles. A full overview of inclusion and exclusion criteria is included in Appendix 2.

Search results were uploaded in Endnote to screen for eligibility and to remove duplicate results. An initial screening based on title and abstract was performed to exclude irrelevant articles or articles that did not meet inclusion criteria. Remaining articles were read in full and excluded if they did not meet inclusion criteria. All articles included in the results section of the review (*n = 85*) were either included from the search or referred to by included articles. A flow diagram of study selection is presented in Appendix 3. One reviewer (LH) collected the following study characteristics from the included articles; first author, species, sex, pain model, treatment, assessment measures (Appendix 4).

All articles included in the result section of the review (*n* = 85) were subjected to a Risk of Bias (RoB) analysis to assess the individual quality of the articles. The SYRCLE RoB tool was used for this evaluation. The items in the RoB tool relate to performance bias, selection bias, attrition bias, detection bias, reporting bias and other biases.^27^ RoB analysis was performed independently by two reviewers (LH, MRM) and their assessment was compared after completion of the analysis. Differences in assessment were discussed and a consensus was reached for each article. Appendix 5 provides RoB analysis for each individual article.

## Results

### Descending serotonergic projections and spinal nociception in adult rodent

The release of serotonin in the spinal dorsal horn activates the various serotonin receptors and thereby modulates nociceptive input to the spinal dorsal horn and the subsequent signal transmission to the brain (i.e. opening or closing the spinal gate). Serotonin has a bidirectional effect on spinal nociceptive processing and, through its receptors, either facilitates or inhibits the incoming nociceptive signal (opening or closing the spinal gate, respectively).^26^

Excitatory receptors in the spinal DH are 5-HT2 (G_q_ coupled), 5-HT3 (ligand-gated ion channel), 5-HT4, 5-HT6 and 5-HT7 (G_s_ coupled). Inhibitory receptors in the spinal DH are 5-HT1 and 5-HT5 (G_i_ coupled).^4,28^ Essentially the net effect of the activation of 5-HT receptors depends on which receptor subtype is activated, either inhibitory (see the next section) or facilitatory (see the Descending serotonergic projections and facilitation of spinal nociception section), to what degree and on which cell type these receptors are located (see Figure 1(b)). If inhibitory 5-HT receptors are expressed on inhibitory interneurons, the net effect is facilitatory, and vice versa.

#### Descending serotonergic projections and inhibition of spinal nociception

Involvement of spinal serotonin in inhibition of nociception has been demonstrated both behaviorally^29,30^ and in electrophysiological studies.^13,31^ Fasmer et al. suggested that serotonin in the spinal cord tonically inhibits reflex based nociception, as serotonin depletion has pronociceptive influences on the tail flick reflex.^32^ Not only spinal, but also systemic and supraspinal administration of serotonin or its precursor 5-hydroxytryptophan (5-HTP) has antinociceptive effects on the tail flick reflex.^33–37^ However, caution should be taken while interpreting tail flick test results as serotonin or its agonists can produce motor effects that possibly influence tail flicks.^36,37^

The inhibitory modulation of 5-HT on nociceptive transmission in the DH is mediated by 5-HT1a, 5-HT1b, 5-HT2a, 5-HT2c, 5-HT3 and 5-HT4 receptors. Evidence for the antinociceptive effect of 5-HT1a has been provided by both behavioral^38–40^ and electrophysiological studies.^39,41–43^ The inhibitory modulation of nociception via the 5-HT1a receptor is likely mediated via a reduced glutamate release from primary afferent terminals (nociceptive afferent pathway, Figure 1(b)). There is, however, one electrophysiological study that shows that spinal 5-HT1a activation does not influence C fiber-evoked spinal field potentials.^44^

The inhibitory effect of spinal 5-HT1b receptor evoked responses of DH neurons was demonstrated through spinal administration of 5-HT1b agonists^13,45,46^ and antagonists.^13^ However, there is some discrepancy in literature about the involvement of 5-HT1b receptors in nociceptive processing as it has been shown that spinal 5-HT1b agonists did not affect reflex-based nociceptive behavior^39,46^ or evoked wide dynamic range (WDR) neuronal responses.^39^ 5-HT1b autoreceptors are expressed on descending serotonergic terminals,^10^ where they inhibit 5-HT release in the DH^47^ (autoreceptor pathway, Figure 1(b)). The described discrepancy on 5-HT1b functionality in descending inhibition of nociception in the spinal cord may be due to the dual effect on both the autoreceptor pathway and the nociceptive afferent pathway, yet this remains to be investigated.

Spinal 5-HT2a receptors are not involved in the inhibition of neuronal responses in DH laminae I and II^44^ nor in nociceptive behavior.^48^ However, involvement of spinal 5-HT2a receptors in nociceptive inhibition cannot be completely ignored as activation of spinal 5-HT2a receptors inhibits c-fiber evoked WDR responses.^13^ This inhibitory effect is likely exerted indirectly via the excitatory interneuron (EXIN) pathway (see Figure 1(b)).

Spinal 5-HT3 agonists produce antinociceptive behaviors^49–51^ whereas spinal 5-HT3 antagonists or 5-HT3 receptor knock-down increase sensitivity to nociceptive stimuli and reduce inhibitory effects of exogenous 5-HT.^49–52^ Electrophysiological studies report inhibitory effects of 5-HT3 receptors.^13,42,53^ Alhaider et al. demonstrated that this inhibitory modulation was mediated by γ-aminobutyric acid (GABA).^49^ Thus, 5-HT3 receptor activation involves the GABAergic inhibitory interneuron (ININ) pathway (see Figure 1(b)). Despite the fact that the expression of 5-HT2c and 5-HT4 in the DH has not been specified, it has been shown that activation of these receptors inhibits C fiber-evoked responses of WDR neurons.^13,44,54^

Little is known about the functionality of the inhibitory 5-HT5a receptor. Although, one would expect, based on its expression on exclusively postsynaptic neurons in the DH, the 5-HT5a receptor to be involved in descending inhibition via both the projection neuron pathway and the EXIN pathway^11^ (see Figure 1(b)).

**Conclusion**: In the healthy adult rodent, serotonin is inhibiting spinal nociceptive neurotransmission. This anti-nociceptive effect is mediated via 5-HT1a, 5-HT1b, 5-HT2c, 5-HT3 and 5-HT4 receptors. Although 5-HT3 is originally excitatory, the presence of this receptor on inhibitory GABAergic interneurons makes its activation resulting also in an inhibitory net-effect on spinal nociceptive neurotransmission.

#### Descending serotonergic projections and facilitation of spinal nociception

Despite overwhelming evidence of antinociceptive effects of centrally administered serotonin in healthy adult rodents (see the previous section), serotonin also may facilitate nociceptive transmission in the spinal dorsal horn. Cai et al. showed that optogenetic activation of serotonergic neurons in the RVM produced persistent sensitization to mechanical and thermal stimuli and suggested that serotonergic neurons in the RVM have a predominant facilitatory role on spinal nociception.^55^

Indeed, pronociceptive behaviors in the healthy rodent have been induced by both spinal and systemic 5-HT1a receptor activation.^46,56,57^ This facilitatory action of the inhibitory 5-HT1a receptor likely involves the GABA ININ pathway (see Figure 1(b)), as 5-HT1a receptors are expressed on GABAergic interneurons.^58^ Bonnefont et al. provided evidence for this by showing that the facilitatory effect of spinal 5-HT1a receptors could be inhibited by the GABA_A_ receptor antagonist bicuculline.^57^ Spinal 5-HT1b receptor activation was shown to increase electrically evoked post-discharge and it was therefore suggested that this probably enhanced the excitability of the GABAergic neurons.^45^

Spinal non-specific 5-HT2a/c agonists and antagonists revealed facilitatory effects on evoked WDR responses.^20^ Whether this facilitatory effect is due to 5-HT2a or 5-HT2c mediated modulation remains to be investigated.

Besides a vast amount of evidence showing inhibitory effects of 5-HT3 receptor activation (via the GABAergic interneurons; see the previous section), activation of this receptor has also been shown to result in pronociceptive modes of action.^59–61^ This facilitatory effect of 5-HT3 receptors can be explained by its expression on primary afferent terminals (nociceptive afferent pathway, see Figure 1(b)), on excitatory interneurons (EXIN pathway, see Figure 1(b)) and/or on projection neurons (projection neuron pathway, Figure 1(b)).^12,14,21,62^ Guo et al. demonstrated that the facilitatory effect of spinal 5-HT3 activation was dose-dependent, as the highest dose of 5-HT3 agonist they used produced antinociceptive effects.^60^

Spinal 5-HT2b and 5-HT7 receptors do not seem to play a key role in the modulation of nociception in the sham-injured rodent^15,44,63^ and therefore might not be involved in serotonergic modulation of nociceptive processing in the DH.

**Conclusion:** Facilitatory effects of descending serotonergic modulation on spinal nociception do exist in the healthy adult rodent and are mediated via activation of 5-HT1a, 5-HT2a/c and 5-HT3 receptors.

#### In conclusion

**Figure 2.**
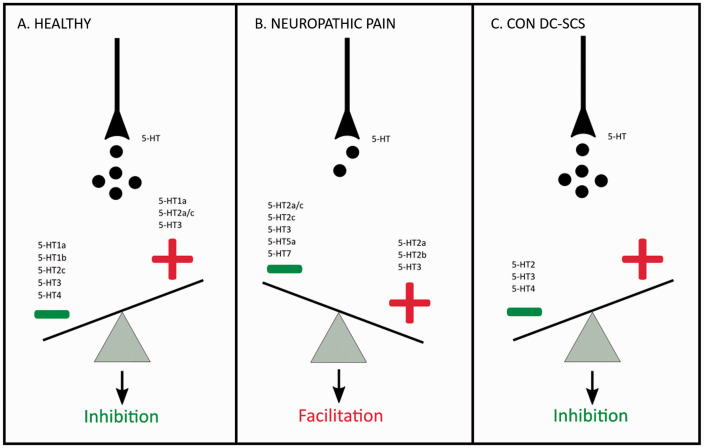
Schematic overview of the effect of descending serotonergic modulation on nociceptive transmission in the DH and the involved receptors in the healthy (a), neuropathic (b) and con-DC-SCS treated (c) rodent. Upon injury (b), 5-HT content in the DH is reduced and 5-HT1b and 5-HT4 receptors lose their inhibitory function. Con-DC-SCS (c) increases 5-HT release in DH. The – symbol means inhibitory mediation, the + symbol means facilitatory modulation.

In the healthy adult rodent there is an overall inhibitory and anti-nociceptive effect of serotonin on spinal nociceptive processing. The ability of descending serotonergic projections to modulate inhibition as well as facilitation of the spinal nociceptive network depends on the receptors involved in relation to their cellular localization. The inhibitory modulation of 5-HT on nociceptive transmission in the DH of the healthy adult rodent is mediated via activation of 5-HT1a, 5-HT1b, 5-HT2c, 5-HT3 and 5-HT4 receptors whereas facilitatory modulation is mediated by 5-HT1a receptors, 5-HT2a/c receptors and 5-HT3 receptors. Spinal 5-HT2b and 5-HT7 receptors are very likely not involved in descending serotonergic modulation of the spinal nociception (see Figure 2(a)).

### Descending serotonergic projections and spinal nociception in chronic neuropathic rodents

Neuropathic pain is caused by a lesion or disease of the somatosensory nervous system.^2^ Injury to peripheral nerves induces a variety of plastic changes in the DH, such as glutamate/NMDA-mediated central sensitization, disinhibition and altered neuron-glia interactions, all contributing to the development and maintenance of chronic neuropathic pain.^64^ The descending serotonergic system is also subject to changes during the development of neuropathic pain.

#### Descending serotonergic projections and inhibition of spinal nociception in neuropathic pain

The descending serotonergic system is subject to changes after nerve injury which disturbs the balance between inhibitory and facilitatory modulation of nociceptive transmission in the spinal DH.^3^ Changes in 5-HT mediated descending inhibition may contribute to the facilitatory effect of serotonin on nociceptive transmission in peripheral neuropathic pain models. In part, this might involve a decrease of 5-HT in the spinal DH as observed in various peripheral nerve injury models.^54,65–67^ Indeed, spinally 5-HT depleted rats are more sensitive upon CCI than non-depleted controls.^68^ On the other hand, levels of spinal 5-HT have also been shown to be increased^69–71^ or unchanged^72,73^ after injury, depending on the neuropathic pain model used.

Alongside the decrease in spinal 5-HT, 5-HT receptors are also subject to changes upon injury. The contribution of spinal 5-HT1 receptors (1a and 1 b) to neuropathic pain remain still somewhat unclear. Although 5-HT1a receptors have been documented to be inhibitory to nociceptive neurotransmission in neuropathic pain models,^38,44,74^ other studies suggest spinal 5-HT1a not to be involved in inhibition^75^ or to lose this inhibitory action.^54^ A similar incomplete picture exists for the spinal 5-HT1b receptor where on the one hand Liu et al. demonstrated a loss of inhibitory function of spinal 5-HT1b receptors using both agonist and antagonist application,^54^ but on the other hand an unchanged inhibitory role was reported.^44^

The spinal 5-HT2c receptors are involved in inhibitory modulation of nociceptive neurotransmission as activation of the spinal 5-HT2c receptor resulted in antiallodynic behaviors in a peripheral SNL injury model.^76^ On the other hand, pharmacological inhibition of spinal 5-HT2c receptors resulted in a loss of effect on 5-HT-evoked WDR neuron activity.^54^ It is therefore possible that SNL injury reduces the inhibitory effects of 5-HT2c on WDR neurons but does not diminish inhibitory 5-HT2c function completely. In the setting of peripheral nerve injury, spinal non-specific 5-HT2a/2c activation seems to switch from a facilitatory effect on nociceptive neurotransmission^20^ to promoting behavioral analgesia.^77,78^ Additionally, these receptors are involved in the antinociceptive effect of SSRIs.^79^

The spinal 5-HT3 receptor loses its inhibitory effect on evoked WDR responses after SNL injury.^54^ At the same time, increased expression of 5-HT3 receptors on primary afferent terminals after CCI of the infraorbital nerve have been suggested by Kim et al.^80^ Increased activation of the nociceptive afferent pathway (see Figure 1(b)) due to increased 5-HT3 expression could explain the reduced inhibitory effect on evoked WDR responses. Additionally, Peters et al. did not report an effect of various doses of i.t. the 5-HT3 antagonist ondansetron on mechanical and thermal hypersensitivity 14 days after SNL injury.^73^ At the same time, intrathecal 5-HT3 agonist m-CPBG application resulted in antiallodynic effects, which is mediated by GABA.^81^ Thus, the inhibitory modulation observed in healthy rats does not seem to be completely abolished.

The 5-HT4 receptor loses its inhibitory function on nociceptive neurotransmission in chronic neuropathic pain models.^44,54^

5-HT5a receptors are involved in mediating antinociceptive effects of exogenously administered 5-HT in the SNL model of chronic neuropathic pain whereas 5-HT5a receptor protein levels are not altered.^74^

Although the 5-HT7 receptor does not seem to play a major role in the modulation of nociceptive transmission in the healthy rodent, both systemic and spinal activation of the 5-HT7 receptor produced antinociception in various neuropathic pain models.^15,16,63^ This related to activation of the GABAergic system^63^ (see Figure 1(b); GABAergic ININ pathway). 5-HT7 receptor expression was shown to be increased in lamina I-II and III-V of the ipsilateral DH in the PSNL mice, whereas 5-HT7 expression is not increased on GABAergic ININs.^15^ Thus, injury might change the proportions of 5-HT7 receptors expressed on different cell types, causing changes in receptor function upon injury.

**Conclusion:** In neuropathic pain, the facilitatory modulation of nonspecific 5-HT2a/c receptors switches to an inhibitory modulation. Serotonergic inhibitory modulation is further mediated by 5-HT2c and 5-HT3 receptors in the chronic neuropathic animal. 5-HT5a receptors are also involved in inhibiting nociceptive signaling in the chronic neuropathic rat whereas its function in the healthy rodent is relatively unknown. The 5-HT7 receptor seems to gain an inhibitory function in modulation of nociceptive signaling upon injury. 5-HT1a and 5-HT1b receptors may also be involved in mediating 5-HTergic descending inhibition, however inconsistencies in the literature do not allow for a definitive conclusion.

#### Descending serotonergic projections and facilitation of spinal nociception in neuropathic pain

Whereas spinal serotonin in healthy nociception has an overall antinociceptive effect (see the Descending serotonergic projections and inhibition of spinal nociception section), peripheral nerve injury seems to result in a more facilitatory effect of spinal serotonin.^82^ This has been shown by several studies on different types of peripheral injury models where spinal or supraspinal serotonin depletion reversed the hypersensitivity induced by the injury induced by the pain model^65,83,84^ and altered evoked responses of WDR neurons to punctate mechanical and heat stimuli.^85^ These analgesic effects of serotonin depletion suggest that serotonin facilitates nociceptive transmission in peripheral neuropathic pain. A study by Vogel et al. reinforces this statement by showing that mice deficient of the serotonin transporter (5-HTT), which is normally expressed on descending serotonergic terminals, do not develop heat hyperalgesia upon CCI of the sciatic nerve. These mice showed a reduced serotonin content in their spinal cord compared to their wild type littermates, which may explain the lack of heat hyperalgesia in these mice. Therefore, the authors conclude that serotonin is involved in the sensitization of nociceptive fibers to heat stimuli.^67^

Spinal 5-HT2a and 5-HT2b receptors seem less important for nociceptive modulation in healthy rodents, but on the contrary their activation is involved in facilitation of C fiber-evoked responses after SNL.^44^ The facilitatory role of spinal 5-HT2a receptors in chronic neuropathic pain has also been demonstrated in other studies.^44,61,86^ In addition, systemic 5-HT2a activation has been shown to result in pronociceptive effects.^58,87^ The facilitatory effects of 5-HT2a and 5-HT2b receptors may be explained by increased expression of these receptors in the spinal DH upon injury.^88^

Spinal 5-HT3 receptors generally become facilitatory upon injury whereas they had an overall inhibitory function in the healthy rodent. This facilitatory effect has been shown in both behavioral^60,89–91^ and electrophysiological studies ^61,80,92^ and might be explained by increased 5-HT3 receptor expression in the DH that was observed after SNL injury.^60^ Although, unchanged expression of 5-HT3 receptors upon SNL injury has also been reported.^73^ As previously mentioned, there is evidence of inhibitory actions of 5-HT3 receptors in chronic pain (the previous section). The discrepancies in literature regarding spinal 5-HT3 receptor function and expression may be related to the type of injury model used and/or experimental design but this issue should be addressed in future studies.

As described in the previous section, 5-HT7 receptors seem to be involved in nociceptive inhibition upon injury. However, one study suggests a facilitatory and pronociceptive effect of 5-HT7 receptors in chronic neuropathic pain. In SNL animals antinociceptive effects of spinal and systemic 5-HT7 inhibition and a reduced 5-HT7 protein content in DH tissue ipsilateral to injury was shown.^93^ These functional differences may be explained by differences in receptor expression and/or protein content as increased 5-HT7 expression was involved in inhibitory antinociceptive effects and the facilitatory pronociceptive effect was observed with reduced 5-HT7 protein content.

**Conclusion**: The balance between facilitation and inhibition of 5-HT tips towards facilitation of nociceptive transmission after injury. Especially 5-HT2a, 5-HT2b and 5-HT3 receptors are involved in mediating the facilitatory effect of 5-HT in the spinal DH. The 5-HT2b receptor seems to gain this function as evidence for its involvement in the modulation of nociceptive transmission in the healthy rodent is lacking.

#### In conclusion

Descending serotonergic projections are mainly facilitating spinal nociception in chronic neuropathic rodents. This pro-nociceptive effect is in contrast to the anti-nociceptive effect of the descending serotonergic project in normal healthy adult rat (see the Descending serotonergic projections and spinal nociception in adult rodent section). This shift from inhibitory to facilitatory mode of action is related to both injury-induced changes in 5-HT content as well as in increased expression of excitatory serotonergic receptors 5-HT2a, 5-HT2b and 5-HT3 receptors and the loss of inhibitory function of 5-HT1b and 5-HT4 receptors (see Figure 2(b)). It should be noted that the use of different preclinical models for chronic neuropathic pain resulted in different outcomes and may explain some of the inconsistencies reported.

### Descending serotonergic projections and spinal nociception: Spinal cord stimulation in chronic neuropathic rodents

In the context of chronic neuropathic pain, spinal cord stimulation of the dorsal columns (DC-SCS) is an important treatment option.^94^ Although still used as a last resort option, it has been shown to result in significant pain relief of patients which did not respond to pharmacological treatment.^95–98^ The mechanism of conventional (con)-DC-SCS in neuropathic pain is based on the Gate Control Theory^99^ and an antidromic effect from the Aβ fibers in the dorsal columns to the nociceptive network in the spinal dorsal horn. The reviewed studies in this section all utilized the con-DC-SCS paradigm. This type of stimulation, also called tonic SCS, delivers equally spaced pulses at a frequency typically ranging between 40–80 Hz.^100^ In an elegant series of experiments it was shown that the pain relieving effect of con-DC-SCS is related to a (major) segmental effect but at the same time a supraspinal loop is stimulated.^101^ How this supraspinal loop is stimulated is not yet exactly known but in this loop the read-out can be monitored by the activity of descending serotonergic fibers and its modulation of spinal nociception.^102^ The involvement of 5-HT in a con-DC-SCS-activated supraspinal loop is further demonstrated by Tazawa et al. In addition, they suggest that 5-HT is less involved in the segmental mechanisms of SCS-induced antinociception but is more related to the onset of a supraspinal loop and mechanism (for review on mechanism of con-DC-SCS see Joosten and Franken, 2020^103^). Con-DC-SCS was shown to induce a decrease of tryptophan hydroxylase (TPH, the 5-HT synthetic enzyme) protein levels in the ipsilateral dorsal quadrant of the lumbar SC but at the same time induced an increased number of TPH positive cells in the dorsal raphe nucleus.^104^ Additionally, con-DC-SCS has been shown to result in increased 5-HT release in the DH in rats with PSNL-induced neuropathic pain.^66^ This increased 5-HT in the superficial DH was observed only in rats responding to SCS treatment (i.e. responders). Therefore, it is concluded that the increased spinal 5-HT is involved in the anti-nociceptive mechanisms of con-DC-SCS.

Besides the con-DC-SCS paradigm, the field of spinal cord stimulation has developed additional stimulation paradigms and has ventured to new stimulation locations (also reviewed in Joosten and Franken, 2020^103^). The use of new paradigms like burst-SCS have been suggested to induce a stronger activation of the supraspinal loop as compared to the use of con-DC-SCS (see the Cortical control of descending 5-HT modulation section) and thus these paradigms may preferentially involve the descending serotonergic system.

#### Descending serotonergic projections and inhibition of spinal nociception with conventional DC-SCS

Studies aimed to characterize which 5-HT receptors are involved in the con-DC-SCS mediated antinociception have revealed that subclinical doses of intrathecal 5-HT2a receptor antagonist and 5-HT4 receptor antagonist counteract the con-DC-SCS-induced analgesic effects.^105^ In addition, subclinical doses of intrathecal 5-HT2 and 5-HT3 receptor agonists enhanced the antinociceptive effect of con-DC-SCS.^105^ Systemic administration of the non-selective 5-HT2a/2c receptor antagonist ketanserin significantly reduces the antinociceptive effect of con-DC-SCS.^106^ This suggests that, in addition to spinal 5-HT2 receptors, either peripheral or supraspinal 5-HT2 receptors are involved in the antinociceptive effect of con-DC-SCS as well.

A loss of inhibitory function of 5-HT4 receptors is involved in development of chronic neuropathic pain (see the Descending serotonergic projections and spinal nociception in chronic neuropathic rodents section) and con-DC-SCS reverses this effect, which results in antinociceptive outcome. As the 5-HT4 receptor is facilitatory, it is therefore likely that its involvement in the analgesic effect of con-DC-SCS is mediated via inhibitory interneurons, the same goes for the 5-HT2a and 5-HT3 receptor. Evidence for the involvement of the GABAergic ININ pathway (see Figure 1(b)) in 5-HT3 mediation of con-DC-SCS induced antinociception is provided by Song et al.^105^ They showed that i.t. application of a sub-effective dose of m-CPBG, a 5-HT3 receptor agonist, enhanced con-DC-SCS pain relieving effect, and then this was eliminated by i.t. application of the GABA_A_ antagonist bicuculline.^105^

Spinal 5-HT1 receptors may also be involved in mediating the analgesic effects of con-DC-SCS because i.t. administration of methysergide reversed the analgesic effects of SCS.^104^ It should be kept in mind that methysergide is a relatively unspecific antagonist that binds both 5-HT1 and 5-HT2 receptor subtypes and thus the exact involvement of spinal 5-HT1 receptor subtype remains unclear.

**Conclusion**: Con-DC-SCS results in antinociception and reverses the pronociceptive effect of the descending serotonergic projections as seen in chronic neuropathic rodents. Con-DC-SCS increases 5-HT release in the DH in chronic neuropathic rodents. This change from a pro- to an antinociceptive effect is mediated via 5-HT2 and 5-HT3 receptors. At the same time con-DC-SCS restores inhibitory function of the 5-HT4 receptor that was lost upon injury.

#### Descending serotonergic projections and facilitation of spinal nociception with conventional DC-SCS

Our search did not result in any articles which demonstrated con-DC-SCS to induce a possible facilitatory or pronociceptive mode of action of descending serotonergic projections on the spinal nociceptive network.

#### In conclusion

Conventional stimulation of the dorsal columns result in increased 5-HT release in the spinal DH of the SCS-responding chronic neuropathic rat. Con-DC-SCS results in antinociception and reverses the pronociceptive effect of the descending serotonergic projections as seen in chronic neuropathic rodents and this involves the 5-HT2, 5-HT3, 5-HT4 receptors (see Figure 2(c)).

## Summary and discussion

### Summary

In the healthy adult rodent, descending serotonergic modulation of nociceptive transmission in the dorsal horn is inhibitory, acting through the 5-HT1a, 5-HT1b, 5-HT2c, 5-HT3 and 5-HT4 receptor.

In chronic pain, the balance tips towards facilitation (and pronociception) which is mediated via injury-induced changes of the descending serotonergic system. Besides a reduced 5-HT content, peripheral nerve injury resulted in upregulated excitatory receptors (5-HT2a, 5-HT2b, 5-HT3) and changes in functionality of spinal 5-HT2a/c, 5-HT2b, 5-HT3, 5-HT4 and 5-HT7 receptors.

Con-DC-SCS restores the balance from pro- to antinociception and this is mediated by 5-HT2, 5-HT3 and 5-HT4 receptors. Similar to the normal healthy situation, the GABAergic ININ pathway is important for mediating the inhibitory and antinociceptive effect of the 5-HT3 receptor in con-DC-SCS induced analgesia.

### Cortical control of descending 5-HT modulation

Although the topic of this systematic review is to collect all data and information on the serotonergic modulation nociception in the spinal cord, (sub)cortical modulation and the role of 5-HT on nociception must not be overlooked. Noxious stimuli activate a variety of brain regions such as the primary and secondary sensory cortices, the anterior cingulate cortex (ACC), prefrontal cortex (PFC), insula, amygdala and thalamus.^1,107,108^ The periaqueductal grey (PAG), located in the midbrain, receives input from these areas and projects to the RVM.^109–111^ The PAG is an important regulator of serotonergic descending modulation through its connections with the RVM.

Besides descending modulation of nociception, 5-HT is involved in ascending modulation of nociception as well. Ascending serotonergic fibers originate from the raphe nuclei and project to a variety of brain areas.^112^ The application of 5-HT or 5-HT receptor agonists and/or antagonists modulates nociception by acting on these brain areas that express serotonin receptors.^34,113,114^

Similarly as to the spinal cord, supraspinal areas or their connectivity are subject to changes upon chronic pain^107,115–118^ and are activated upon DC-SCS with both conventional and burst paradigms.^102,119^

Clearly, effects of serotonergic drugs and/or neuromodulatory treatments on supraspinal brain regions that are involved in nociception and pain and that express serotonin receptors must not be overlooked in the development of new treatments.

### Pharmacological interventions for chronic pain: Role for serotonergic drugs?

Pharmacological interventions in the treatment of neuropathic pain are often complicated as they are accompanied by substantial side-effects which cause them to be discontinued. Currently, pharmacological treatment paradigms include the use of tricyclic antidepressants such as amitriptyline and serotonin-noradrenalin reuptake inhibitors (SNRIs) such as duloxetine.^120,121^

Since 5-HT receptors are subject to change after injury, specific targeting of these receptors is recommended for better treatment of neuropathic pain. Since the 5-HT2b receptor does not seem to be involved in nociceptive modulation in the healthy rodent but does exert a facilitatory role in neuropathic rodents, this receptor may be an important candidate for pharmacological treatment and the development of a selective antagonist. As the 5-HT5a and 5-HT7 receptors seem to show increased inhibitory function upon injury, the activation of these receptors with the use of very specific agonists may flip the overall balance from pro- to antinociception.

5-HT also plays a role in nerve injury-induced long term potentiation of synapses within the spinal cord that contribute to chronic pain,^122^ in one way by transforming silent glutamatergic synapses in the spinal dorsal horn into functional synapses.^123^ Future studies on elucidating the exact involvement of 5-HT in this transformation process and LTP is needed to further understand the mechanism of action underlying use of serotonergic drug or neuromodulatory treatments in chronic pain.

### Combination of serotonergic drugs and SCS: Rescuing non-responders?

Unfortunately, of the patients that receive con-DC-SCS, about 30% of patients to not experience clinically relevant pain relief and are classified as nonresponders.^124^ The combination of serotonergic drug treatment and con-DC-SCS might also lead to a better treatment for neuropathic pain patients. Preclinical work from Song et al. showed that increased 5-HT release in the spinal cord was only observed in rats responding to con-DC-SCS and that the combination with subclinical doses of intrathecally applied serotonergic drugs could enhance the SCS-induced analgesic effect and turn nonresponders to con-DC-SCS into responders. Other examples of turning nonresponders into responders with the use of sub-effective drug dose application are ketamine^124^ or the GABA_B_ antagonist baclofen.^125^ Because 5-HT is clearly involved in mediating con-DC-SCS-induced analgesia, combinational therapy of serotonergic drugs and SCS may potentially be of use in rescuing patients that do not respond to SCS treatment alone. Antidepressants have been used as adjuvant therapy in both preclinical and clinical studies, resulting in an enhanced effectivity of con-DC-SCS^126^ and improvements on McGill pain questionnaire and willingness to repeat SCS surgery,^127^ respectively. Because of the close entanglement with the GABAergic system, modulation of 5-HT3 receptors might be of specific interest.

### New SCS-paradigms: A more prominent role of descending serotonergic projections and spinal nociception?

As briefly mentioned in the Descending serotonergic projections and spinal nociception: Spinal cord stimulation in chronic neuropathic rodents section, there are recently developed and new SCS stimulation paradigms which are now tested in neuropathic pain patients such as burst and high frequency SCS (reviewed in Heijmans and Joosten, 2020^128^). Burst-SCS is a paradigm that delivers periodic bursts of multiple pulses to the dorsal column. Based on EEG and imaging studies^119,129,130^ and the behavioral observation that burst-SCS has a delayed onset of efficacy and a prolonged duration of efficacy after discontinuation of stimulation, when compared to con-SCS,^131^ it is suggested that burst-SCS activates both the medial and lateral ascending spinothalamic tract whereas con-DC-SCS only seems to activate the lateral.^128^ The medial pathway is known to be involved in processing emotional, affective components of pain and engages the RVM and PAG in a descending feedback loop to the spinal DH.^64^ Burst-SCS activates the anterior cingulate cortex and amygdala (among other brain areas) to a higher extent with than con-DC-SCS and these areas provide output to the RVM and the PAG.^119^ As the RVM and PAG are important brain areas in serotonergic descending modulation,^109^ it can be speculated that serotonin plays a role in the analgesic mechanisms of burst-SCS, likely even more than with con-DC-SCS and it will be meaningful to investigate this. Gaining a better understanding of the involvement of the descending serotonergic system in the underlying mechanisms of new SCS paradigms like burst-SCS is therefore of utmost importance.

### Future directions

#### Resolving knowledge gaps

Besides the relatively extensive amount of research on the involvement of serotonin in nociceptive transmission in the healthy rodent and in the neuropathic pain rodent, there remain some important gaps in the current knowledge on the topic that should be addressed in future studies. Firstly, further clarification on the expression of the 5-HT receptors (see Table 1) on the different cell types within the dorsal horn may help the interpretation of behavioral or electrophysiological results and eliminate speculation both in the healthy rodent and after the induction of neuropathic pain. Secondly, present literature on changes in 5-HT expression in the DH after injury is rather conflicting, which may be due to different pain models used. Studies aimed at, or including, the evaluation of changes in 5-HT content or descending serotonergic terminals in the DH after injury may provide more clarity. Thirdly, identifying changes in 5-HT receptor expression and function upon injury will help pinpoint pharmacological targets for a more successful use of serotonergic drugs in the treatment of chronic neuropathic pain.

#### Limitations of included studies

There are some limitations to the studies included in this review that must be addressed. First of all, the use of different pain models and the differences in experimental design and lack of standardization between studies complicates the interpretation of results. Many aspects may affect the net effect of the bidirectional serotonergic modulation such as dose of serotonergic agent, drug administration route (intrathecal, systemic, intraplantar or intraventricular), as well as the time point of testing after induction of the neuropathy (e.g. 7 DPI vs 14 DPI). These should all be considered when comparing the results of different preclinical studies.

Another important point complicating the interpretation of the reviewed literature is that many of the serotonergic drugs used in the studies are not exclusively binding to one receptor subtype. The 5-HT1a agonist 8-OH-DPAT also has affinity for the 5-HT7 receptor.^132^ Similarly, many studies designate ketanserin as a 5-HT2a antagonist whereas it also has affinity for other 5-HT2 receptors. Furthermore, studies using the nonspecific serotonergic drugs methysergide or methiothepin that claim a receptor specific effect must be interpreted with extreme caution. At present, more selective serotonergic drugs are being developed, hopefully this will resolve this problem and provide very detailed and exclusive outcomes.

Lastly, the utilization of behavioral tests should be critically evaluated. Many of the behavioral studies included in this review evaluate evoked pain using reflex based tests, such as the tail flick test, hot plate test and paw withdrawal tests. To assess spontaneous pain or evaluate the effects of interventions that involve supraspinal mechanisms known to be involved in pain, future studies should extend the behavioral test repertoire to include not only evoked pain tests but also tests related to cognitive and emotional aspects of pain such as Conditioned Placed Preference^133^ or the Mechanical Conflict Avoidance System.^134^ This then could also employ diffuse noxious inhibitory controls as a way of evaluating descending inhibition and serotonergic involvement herein.^135–139^ Serotonin is also involved in the regulation of locomotion.^140^ The use of serotonergic drugs at concentrations that induce motor effects may lead to incorrect interpretation of test results. Future studies utilizing serotonergic drugs should include some form of locomotion testing or incorporate a pilot study to select a correct dosage that does not induce locomotion effects.

#### Risk of bias

The RoB analysis was performed on all studies included in the review (Appendix 5). Results showed overall good reporting of baseline characteristics and studies were generally free of selective reporting bias. Randomization (sequence generation, allocation concealment, random housing and random outcome collection) and blinding during the study as well as analysis are not well reported on. Incomplete outcome data was a high risk of bias in quite a lot of articles and should be reported more meticulously. Other potential biases such as conflict of interest and general study design were generally not well reported.

## Conclusion

In the healthy rodent, descending serotonergic modulation of nociceptive transmission in the dorsal horn is inhibitory, acting via the 5-HT1a, 5-HT1b, 5-HT2c, 5-HT3 and 5-HT4 receptor. In chronic neuropathic pain, the balance tips towards facilitation which is mediated by the 5-HT2a, 5-HT2b and 5-HT3 receptor. Con-DC-SCS restores this balance again to an inhibitory mode, which is mediated by 5-HT2, 5-HT3 and 5-HT4 receptors. Future studies with use of new SCS paradigms might benefit from additional use of very selective and sub-effective dose of drugs modulating the serotonergic descending pathway.

## Supplemental Material

sj-pdf-1-mpx-10.1177_17448069211043965 - Supplemental material for A systematic review on descending serotonergic projections and modulation of spinal nociception in chronic neuropathic pain and after spinal cord stimulation Click here for additional data file. Supplemental material, sj-pdf-1-mpx-10.1177_17448069211043965 for A systematic review on descending serotonergic projections and modulation of spinal nociception in chronic neuropathic pain and after spinal cord stimulation by Lonne Heijmans, Martijn R Mons and Elbert A Joosten in Molecular Pain

sj-pdf-2-mpx-10.1177_17448069211043965 - Supplemental material for A systematic review on descending serotonergic projections and modulation of spinal nociception in chronic neuropathic pain and after spinal cord stimulation Click here for additional data file. Supplemental material, sj-pdf-2-mpx-10.1177_17448069211043965 for A systematic review on descending serotonergic projections and modulation of spinal nociception in chronic neuropathic pain and after spinal cord stimulation by Lonne Heijmans, Martijn R Mons and Elbert A Joosten in Molecular Pain

sj-pdf-3-mpx-10.1177_17448069211043965 - Supplemental material for A systematic review on descending serotonergic projections and modulation of spinal nociception in chronic neuropathic pain and after spinal cord stimulation Click here for additional data file. Supplemental material, sj-pdf-3-mpx-10.1177_17448069211043965 for A systematic review on descending serotonergic projections and modulation of spinal nociception in chronic neuropathic pain and after spinal cord stimulation by Lonne Heijmans, Martijn R Mons and Elbert A Joosten in Molecular Pain

sj-pdf-4-mpx-10.1177_17448069211043965 - Supplemental material for A systematic review on descending serotonergic projections and modulation of spinal nociception in chronic neuropathic pain and after spinal cord stimulation Click here for additional data file. Supplemental material, sj-pdf-4-mpx-10.1177_17448069211043965 for A systematic review on descending serotonergic projections and modulation of spinal nociception in chronic neuropathic pain and after spinal cord stimulation by Lonne Heijmans, Martijn R Mons and Elbert A Joosten in Molecular Pain

sj-pdf-5-mpx-10.1177_17448069211043965 - Supplemental material for A systematic review on descending serotonergic projections and modulation of spinal nociception in chronic neuropathic pain and after spinal cord stimulation Click here for additional data file. Supplemental material, sj-pdf-5-mpx-10.1177_17448069211043965 for A systematic review on descending serotonergic projections and modulation of spinal nociception in chronic neuropathic pain and after spinal cord stimulation by Lonne Heijmans, Martijn R Mons and Elbert A Joosten in Molecular Pain
