# Supplementary material for: A systematic review on descending serotonergic projections and modulation of spinal nociception in chronic neuropathic pain and after spinal cord stimulation
Source: Mol Pain. 2021 Oct 18;17:17448069211043965. doi: 10.1177/17448069211043965 (PMC8527581; doi:10.1177/17448069211043965)
Supplement: sj-pdf-1-mpx-10.1177_17448069211043965 - Supplemental material for A systematic review on descending serotonergic projections and modulation of spinal nociception in chronic neuropathic pain and after spinal cord stimulation [file sj-pdf-1-mpx-10.1177_17448069211043965.pdf]

Appendix 1 : Search key words and strategy

Key words for the search were subdivided in the following topics: Serotonin, pain, area and neuromodulation. [] used below indicate the topic and these were not included in the search.

Search 1: Serotonin + Pain + Area
Search 2: Serotonin + Pain + Area + Neuromodulation

PUBMED: searched 15-04-2020

[Serotonin]
(serotonin receptor[MeSH Terms]) OR (serotonin[MeSH Terms]) OR (5 ht1 serotonin receptor[MeSH Terms]) OR (1a receptor, serotonin[MeSH Terms]) OR (1b receptor, serotonin[MeSH Terms]) OR (receptor, serotonin, 5 ht1d[MeSH Terms]) OR (5 ht2 receptor, serotonin[MeSH Terms]) OR (2a receptor, serotonin[MeSH Terms]) OR (2b receptor, serotonin[MeSH Terms]) OR (2c receptor, serotonin[MeSH Terms]) OR (receptor, serotonin 3[MeSH Terms]) OR (receptor, serotonin 4[MeSH Terms]) OR (5-HT5 receptor[Supplementary concept]) OR (5-HT6 receptor[Supplementary Concept]) OR (5-ht7 receptor[Supplementary Concept]) OR

(3-2-aminoethyl-1h-indol-5-ol) OR (5 hydroxytryptamine) OR (5-ht) OR (5-hydroxytryptamine) OR (enteramine) OR (hippophaine) OR (hydroxytryptamine) OR (serotonin) OR (3 2 aminoethyl 5 hydroxyindole) OR (3 2 aminoethyl 5 indolol) OR (3 beta aminoethyl 5 hydroxyindole) OR (5 hydroxy 3 beta aminoethyl indole) OR (5 hydroxy tryptamine) OR (5 hydroxytryptamin) OR (alpha 5 hydroxytryptamine) OR (d,s substance) OR (ds substance) OR (hydroxy tryptamine) OR (serotin) OR (serotonine) OR (thrombocytin) OR (thrombotonin) OR

(5-hydroxytryptamine receptor) OR (serotonin receptor) OR (tryptamine receptor) OR (serotonergic receptor) OR (serotoninergic receptor) OR

(5 hydroxytryptamine 1 receptor) OR (5 ht1 receptor) OR (5-ht1 receptor) OR (5-ht1 serotonin receptor) OR (serotonin 5 ht1 receptor) OR (serotonin 5-ht1 receptor) OR (serotonin receptor s1) OR (serotonin s1 receptor) OR (serotoninergic s1 receptor) OR (5 ht1a receptor) OR (5 hydroxytryptamine 1a receptor) OR (5-ht1a receptor) OR (5-ht1a serotonin receptor) OR (5-ht1a receptor serotonin) OR (5-hydroxytryptamine 1a receptor) OR (serotonin 1a receptor) OR

(5 ht1b receptor) OR (5 ht1dbeta receptor) OR (5 hydroxytryptamine 1b receptor) OR (5-ht1b receptor) OR (5-ht1dbeta receptor) OR (5-hydroxytryptamine 1b receptor) OR (serotonin 1b receptor) OR (serotonin 1dbeta receptor) OR

(5 ht1d receptor) OR (5 hydroxytryptamine 1d receptor) OR (5 hydroxytryptamine1d receptor) OR (5-ht1d receptor) OR (5-ht1dalpha receptor) OR (5-hydroxytryptamine1d receptor) OR (hydroxytryptamine1d) OR (serotonin 1d alpha receptor) OR (serotonin 1d receptor) OR (serotonin 1dalpha receptor) OR

(5 ht2 receptor) OR (5-ht-2 receptor) OR (5-ht2 serotonin receptors) OR (5 hydroxytryptamine 2 receptor) OR (serotonin 2 receptors) OR (serotonin s2 receptors) OR (serotonin-2 receptors) OR (serotoninergic s2 receptor) OR

(5-ht2a receptor) OR (5 hydroxytryptamine 2a receptor) OR (serotonin 2a receptor) OR

(5-ht2b receptor) OR (5 hydroxytryptamine 2b receptor) OR (5-ht2b receptor) OR (5-hydroxytryptamine 2b receptor) OR (serotonin 2b receptor) OR

(5 hydroxytryptamine 2c receptor) OR (5 hydroxytryptamine type 2c receptor) OR (5-ht2c receptor) OR (5-hydroxytryptamine type 2c receptor) OR (serotonin 2c receptor) OR

(5ht3a receptor) OR (5ht3b receptor) OR (5 hydroxytryptamine 3 receptor) OR (5 hydroxytryptamine 3a receptor) OR (5 hydroxytryptamine 3b receptor) OR (5-ht3 receptor) OR (5-hydroxytryptamine-3 receptor) OR (serotonin 3 receptor) OR (serotonin receptor s3) OR (serotonin s3 receptor) OR

(5 hydroxytryptamine 4 receptor) OR (5-ht4 receptor) OR (5-ht4l receptor) OR (5-ht4s receptor) OR (5-hydroxytryptamine-4 receptor) OR (serotonin 4 receptor) OR (serotonin s4 receptor) OR

(5-HT5 receptor) OR (5-hydroxytryptamine 5 receptor) OR (5-hydroxytryptamine 5a receptor) OR (5-hydroxytryptamine 5b receptor) OR (5HT5 receptor) OR (serotonin 5A receptor) OR (serotonin receptor 5A) OR (5-HT5A receptor) OR (serotonin 5B receptor) OR (5-HT5B receptor) OR

(5-HT6 receptor) OR (5-hydroxytryptamine 6 receptor) OR (serotonin 6 receptor) OR

(5-HT7 receptor) OR (5-hydroxytryptamine-7 receptor) OR (serotonin 7 receptor)

AND

[Pain]
(pain[MeSH Terms:noexp) OR (nociceptors[MeSH Terms]) OR (nociception[MeSH Terms]) OR (small fiber neuropathy[MeSH Terms]) OR (sciatic neuropathy[MeSH Terms]) OR (chronic pain[MeSH Terms]) OR (neuropathic pain[MeSH Terms:noexp]) OR (peripheral nerve injuries[MeSH Terms]) OR (central sensitization[MeSH Terms]) OR (low back pain[MeSH Terms]) OR (failed back surgery syndrome[MeSH Terms]) OR (complex regional pain syndromes[MeSH Terms]) OR (crps type i[MeSH Terms]) OR (crps type ii[MeSH Terms]) OR (painful diabetic neuropathy[MeSH Terms]) OR (intractable pain[MeSH Terms]) OR (radiculopathy[MeSH Terms]) OR

(ache) OR (acute pain) OR (burning pain) OR (crushing pain) OR (deep pain) OR (lightning pain) OR (migratory pain) OR (pain) OR (radiating pain) OR (splitting pain) OR (treatment related pain) OR (mechanonociceptor) OR (nociceptive neuron) OR (nociceptive receptor) OR (nociceptor) OR (nocireceptor) OR (pain receptor) OR (nociception) OR (nociperception) OR (pain perception) OR (pain sensation) OR (pain sense) OR (pain sensitivity) OR

(small fiber neuropathy) OR (small fibre neuropathy) OR (small nerve fiber neuropathy) OR

(ischias) OR (ischiatic pain) OR (lesion of sciatic nerve) OR (sciatic nerve disease) OR (sciatic nerve injury) OR (sciatic nerve lesion) OR (sciatic nerve neuralgia-neuritis) OR (sciatic nerve palsy) OR (sciatic neuritis) OR (sciatic neuropathy) OR (sciatic pain) OR (sciatica) OR

(chronic intractable pain) OR (chronic pain) OR (widespread chronic pain) OR

(atypical neuralgia) OR (iliohypogastric nerve neuralgia) OR (ilioinguinal neuralgia) OR (nerve pain) OR (neuralgia) OR (neurodynia) OR (neuropathic pain) OR (paroxysmal nerve pain) OR (perineal neuralgia) OR (stump neuralgia) OR (supraorbital neuralgia) OR (vidian neuralgia) OR

(peripheral nerve damage) OR (peripheral nerve injury) OR (peripheral nerve trauma) OR

(central nervous system sensitization) OR (central sensitization) OR

(chronic low back pain) OR (loin pain) OR (low back ache) OR (low back pain) OR (low backache) OR (lowback pain) OR (lower back pain) OR (lumbago) OR (lumbal pain) OR (lumbal syndrome) OR (lumbalgia) OR (lumbar pain) OR (lumbar spine syndrome) OR (lumbar syndrome) OR (lumbodynia) OR (lumbosacral pain) OR (lumbosacral root syndrome) OR (lumbosacroiliac strain) OR (mechanical low back pain) OR (postural low back pain) OR (recurrent low back pain) OR

(failed back surgery syndrome) OR (failed back surgery) OR (failed back syndrome) OR (FBSS) OR (post-laminectomy syndrome) OR (postlaminectomy syndrome) OR

(complex regional pain syndrome) OR (crps) OR

(algodystrophia) OR (algodystrophic syndrome) OR (algodystrophies) OR (algodystrophy) OR (algoneurodystrophy) OR (cervical sympathetic dystrophy) OR (complex regional pain syndrome 1) OR (complex regional pain syndrome type 1) OR (complex regional pain syndrome type I) OR (CRPS 1) OR (CRPS I) OR (CRPS type 1) OR (crps type i) OR (CRPS-I) OR (neuralgic shoulder amyotrophy) OR (posttraumatic dystrophy) OR (post-traumatic dystrophy) OR (posttraumatic osteopenia) OR (posttraumatic osteoporosis) OR (reflex sympathetic dystrophy) OR (reflex sympathetic dystrophy syndrome) OR (rsd) OR (rsds) OR (shoulder-hand syndrome) OR (shoulder-hand syndromes) OR (Sudeck atrophy) OR (Sudeck disease) OR (Sudeck dystrophy) OR (Sudeck Leriche syndrome) OR (Sudeck syndrome) OR (Sudeck's atrophy) OR (sudeks atrophy) OR (sudek's atrophy) OR (Suedeck dystrophy) OR (Suedeck reflex dystrophy) OR (sympathetic reflex dystrophia) OR (sympathetic reflex dystrophy) OR

(causalgia) OR (causalgia syndrome) OR (complex regional pain syndrome type ii) OR (CRPS 2) OR (CRPS II) OR (CRPS type 2) OR (crps type ii) OR (CRPS-II) OR (deafferentation pain) OR

(asymmetric diabetic proximal motor neuropathy) OR (diabetes neuropathy) OR (diabetic amyotrophy) OR (diabetic asymmetric polyneuropathy) OR (diabetic autonomic neuropathy) OR (diabetic mononeuropathy) OR (diabetic mononeuropathy simplex) OR (diabetic neuralgia) OR (diabetic neuritis) OR (diabetic neuropathy) OR (diabetic peripheral neuropathy) OR (diabetic polyneuritis) OR (diabetic polyneuropathy) OR (painful diabetic neuropathy) OR (symmetric diabetic proximal motor neuropathy) OR

(intractable pain) OR (refractory pain) OR

(cervical radiculopathy) OR (nerve root avulsion) OR (nerve root compression) OR (Nerve root disease) OR (nerve root disorder) OR (nerve root inflammation) OR (neuroradiculitis) OR (polyneuroradiculitis) OR (Polyradiculopathy) OR (radiculalgia) OR (radicular neuralgia) OR (Radicular neuropathy) OR (radicular pain) OR (radiculitis) OR (radiculopathy) OR (spinal radiculitis) OR

(Discogenic pain) OR

(allodynia) OR (Cold allodynia) OR (Cold hyperalgesia) OR (Heat allodynia) OR (Heat hyperalgesia) OR (hyperalgesia) OR (hyperalgia) OR (hyperpathia) OR (mechanical allodynia) OR (mechanical

hyperalgesia) OR (mechanical hyperalgia) OR (mechanical hyperpathia) OR (tactile allodynia) OR (thermal allodynia) OR (thermal hyperalgesia) OR (thermal hyperalgia) OR (thermal hyperpathia) OR

(Experimental neuropathic pain) OR (Experimentally induced neuropathic pain) OR (Experimental pain) OR (Experimentally induced pain) OR (nociceptive pain) OR (somatic pain) OR (tissue pain) OR (Experimental diabetic neuropathy) OR (Experimentally induced diabetic neuropathy) OR (streptozocin-induced diabetic neuropathy) OR (streptozotocin-induced diabetic peripheral neuropathy) OR (STZ-diabetic neuropathy) OR (STZ-induced diabetic neuropathy) OR (STZ-induced diabetic peripheral neuropathy) OR (spared nerve injury) OR (partial sciatic nerve injury) OR (chronic constriction injury) OR (spinal cord injury) OR (spinal nerve ligation) OR (spinal hemisection)

AND

[Area]
(spinal cord[MeSH Terms:noexp]) OR (dorsal horn[MeSH Terms:noexp]) OR (substantia gelatinosa[MeSH Terms]) OR (interneurons[MeSH Terms:noexp]) OR (periaqueductal gray[MeSH Terms]) OR (nucleus raphe magnus[MeSH Terms]) OR (somatosensory cortex[MeSH Terms]) OR (spinothalamic tract[MeSH Terms]) OR (dorsal root ganglion[MeSH Terms]) OR (Posterior horn cells[MeSH Terms]) OR

(cervical cord) OR (cervical medulla) OR (coccygeal cord) OR (conus medullaris) OR (conus terminalis) OR (cornu laterale) OR (lateral horn) OR (lumbar cord) OR (medulla spinalis) OR (medullary cone) OR (myelon) OR (pars cervicalis medullae spinalis) OR (pars lumbalis medullae spinalis) OR (pars thoracica medullae spinalis) OR (sacral cord) OR (sacral spine) OR (spinal cord) OR (spinal marrow) OR (spinal medulla) OR (thoracic cord) OR

(clarke columns) OR (clarke nucleus) OR (clarkes columns) OR (clarke's columns) OR (clarkes nucleus) OR (clarke's nucleus) OR (columna posterior substantiae griseae) OR (cornu posterius substantiae griseae) OR (dorsal horn) OR (marginal nucleus) OR (nucleus dorsali) OR (nucleus dorsalis) OR (posterior horn) OR (posterior horn of spinal cord) OR (posterior spinal horn) OR (spinal cord dorsal horn) OR (spinal cord posterior horn) OR (spinal dorsal column) OR (spinal dorsal horn) OR (spinal posterior column) OR (zona spongiosa) OR

(lamina 2) OR (lamina ii) OR (rolando substance) OR (rolando substantia gelatinosa) OR (substantia gelatinosa) OR (substantia gelatinosa medullae spinalis) OR (substantia gelatinosa of rolando) OR (substantia gelatinosa rolandi) OR

(intercalary neuron) OR (intercalated nerve cell) OR (intercalated neuron) OR (interneuron) OR (relay nerve cell) OR (relay neuron) OR

(central gray substance of midbrain) OR (central periaqueductal gray) OR (griseum centrale) OR (griseum centrale mesencephali) OR (griseum centrale mesencephalus) OR (mesencephalic central gray) OR (midbrain central gray) OR (periaqueductal gray) OR (periaqueductal gray matter) OR (substantia grisea centralis) OR (substantia grisea centralis mesencephali) OR

(nucleus raphe magnus) OR (nucleus raphe ponti) OR (nucleus raphe pontis) OR (pontine raphe nucleus) OR (raphe magnus) OR (Raphe Magnus Nucleus) OR (raphe ponti) OR (raphe pontis) OR (Raphe Pontis Nucleus) OR

(anterior parietal cortex) OR (area S1) OR (area S2) OR (barrel cortex) OR (first somatosensory area) OR (first somatosensory cortex) OR (gyrus postcentralis) OR (neurosensory field) OR (postcentral gyrus) OR (primary somatic sensory area) OR (primary somatosensory area) OR (primary somatosensory cortex)

OR (S1 area) OR (second somatic sensory area) OR (second somatosensory area) OR (second somatosensory cortex) OR (secondary sensory cortex) OR (secondary somatosensory area) OR (secondary somatosensory cortex) OR (si cortex) OR (somate sensory cortex) OR (somatic sensory cortex) OR (somatosensory area) OR (somatosensory area S1) OR (somatosensory area S2) OR (somatosensory cortex) OR (somatosensory S1 area) OR

(spinothalamic tract) OR (spino-thalamic tract) OR (tractus spinothalamicus) OR

(dorsal root ganglion) OR (dorsal root spinal ganglion) OR (ganglion spinale) OR (spinal dorsal root ganglion) OR (spinal ganglion) OR (spine ganglion) OR

(dorsal horn cell) OR (dorsal horn neuron) OR (posterior horn cell) OR (posterior horn neuron) OR

(rostral ventromedial medulla)

AND

[Neuromodulation]
(electrical stimulation[MeSH Terms:noexp]) OR (spinal cord stimulation[MeSH Terms]) (percutaneous electrical nerve stimulation[MeSH Terms]) OR (transcutaneous electrical nerve stimulation[MeSH Terms]) OR (pulsed radiofrequency treatment[MeSH Terms]) OR (deep brain stimulation[MeSH Terms]) OR (transcranial magnetic stimulation[MeSH Terms]) OR (transcranial direct current stimulation[MeSH Terms]) OR

(Dorsal column stimulation) OR (SCS) OR (Dorsal root ganglion stimulation) OR (PENS) OR (PRF) OR (DBS) OR (rTMS) OR (TMS) OR (brain stimulation) OR (motor cortex stimulation) OR (electromagnetic stimulation) OR (cortex stimulation) OR

(electric field stimulation) OR (electric stimulation) OR (electrical stimulation) OR (electrostimulation) OR (electrostimulus) OR (galvanostimulation) OR

(spinal cord stimulation) OR (spinal stimulation) OR

(analgesic cutaneous electrostimulation) OR (electroanalgesia) OR (percutaneous electric nerve stimulation) OR (percutaneous electrical nerve stimulation) OR (percutaneous electrical neuromodulation) OR (percutaneous electrical) OR (percutaneous neuromodulation therapy) OR (tens) OR (transcutaneous electric nerve stimulation) OR (transcutaneous electric stimulation) OR (transcutaneous electrical nerve stimulation) OR (transcutaneous electrical stimulation) OR (transcutaneous electrostimulation) OR (transcutaneous nerve stimulation) OR (transdermal electrostimulation) OR

(nerve stimulus) OR (neurostimulation) OR (peripheral nerve stimulation) OR (nerve stimulation) OR

(pulsed radio frequency treatment) OR (pulsed radiofrequency treatment) OR

(brain depth stimulation) OR (brain excitation) OR (brain stimulation) OR (brain stimulus) OR (deep brain stimulation) OR (electrical brain stimulation) OR (electrical stimulation of the brain) OR

(repetitive transcranial magnetic stimulation) OR (transcranial magnetic stimulation) OR (paired pulse transcranial magnetic stimulation) OR (repetitive transcranial magnetic stimulation) OR (single pulse transcranial magnetic stimulation) OR

(anodal stimulation tdcs) OR (cathodal stimulation tdcs) OR (repetitive transcranial electrical stimulation) OR (tDCS) OR (transcranial alternating current stimulation) OR (transcranial direct current stimulation) OR (transcranial electrical stimulation) OR (transcranial random noise stimulation)

MEDLINE (ovid): searched 15-04-2020

[Serotonin]
exp serotonin 5A receptor/ or exp serotonin 4 receptor/ or exp serotonin 2A receptor/ or exp serotonin receptor/ or exp serotonin 2B receptor/ or exp serotonin 2 receptor/ or exp serotonin/ or exp serotonin 2C receptor/ or exp serotonin 1A receptor/ or exp serotonin 7 receptor/ or exp serotonin 3 receptor/ or exp serotonin 1 receptor/ or exp serotonin 1B receptor/ or exp serotonin 1D receptor/ or exp serotonin 6 receptor/ or exp serotonin 5 receptor/ or

(3-2-aminoethyl-1h-indol-5-ol or 5 hydroxytryptamine or 5-ht or 5-hydroxytryptamine or enteramine or hippophaine or hydroxytryptamine or serotonin or 3 2 aminoethyl 5 hydroxyindole or 3 2 aminoethyl 5 indolol or 3 beta aminoethyl 5 hydroxyindole or 5 hydroxy 3 beta aminoethyl indole or 5 hydroxy tryptamine or 5 hydroxytryptamin or alpha 5 hydroxytryptamine or d,s substance or ds substance or hydroxy tryptamine or serotin or serotonine or thrombocytin or thrombotonin or 5-hydroxytryptamine receptor or serotonin receptor or tryptamine receptor or serotonergic receptor or serotoninergic receptor or 5 hydroxytryptamine 1 receptor or 5 ht1 receptor or 5-ht1 receptor or 5-ht1 serotonin receptor or serotonin 5 ht1 receptor or serotonin 5-ht1 receptor or serotonin receptor s1 or serotonin s1 receptor or serotoninergic s1 receptor or 5 ht1a receptor or 5 hydroxytryptamine 1a receptor or 5-ht1a receptor or 5-ht1a serotonin receptor or 5-ht1a receptor serotonin or 5-hydroxytryptamine 1a receptor or serotonin 1a receptor or 5 ht1b receptor or 5 ht1dbeta receptor or 5 hydroxytryptamine 1b receptor or 5-ht1b receptor or 5-ht1dbeta receptor or 5-hydroxytryptamine 1b receptor or serotonin 1b receptor or serotonin 1dbeta receptor or 5 ht1d receptor or 5 hydroxytryptamine 1d receptor or 5 hydroxytryptamine1d receptor or 5-ht1d receptor or 5-ht1dalpha receptor or 5-hydroxytryptamine1d receptor or hydroxytryptamine1d or serotonin 1d alpha receptor or serotonin 1d receptor or serotonin 1dalpha receptor or 5 ht2 receptor or 5-ht-2 receptor or 5-ht2 serotonin receptors or 5 hydroxytryptamine 2 receptor or serotonin 2 receptors or serotonin s2 receptors or serotonin-2 receptors or serotoninergic s2 receptor or 5-ht2a receptor or 5 hydroxytryptamine 2a receptor or serotonin 2a receptor or 5-ht2b receptor or 5 hydroxytryptamine 2b receptor or 5-ht2b receptor or 5-hydroxytryptamine 2b receptor or serotonin 2b receptor or 5 hydroxytryptamine 2c receptor or 5 hydroxytryptamine type 2c receptor or 5-ht2c receptor or 5-hydroxytryptamine type 2c receptor or serotonin 2c receptor or 5ht3a receptor or 5ht3b receptor or 5 hydroxytryptamine 3 receptor or 5 hydroxytryptamine 3a receptor or 5 hydroxytryptamine 3b receptor or 5-ht3 receptor or 5-hydroxytryptamine-3 receptor or serotonin 3 receptor or serotonin receptor s3 or serotonin s3 receptor or 5 hydroxytryptamine 4 receptor or 5-ht4 receptor or 5-ht4l receptor or 5-ht4s receptor or 5-hydroxytryptamine-4 receptor or serotonin 4 receptor or serotonin s4 receptor or 5-HT5 receptor or 5-hydroxytryptamine 5 receptor or 5-hydroxytryptamine 5a receptor or 5-hydroxytryptamine 5b receptor or 5HT5 receptor or serotonin 5A receptor or serotonin receptor 5A or 5-HT5A receptor or serotonin 5B receptor or 5-HT5B receptor or 5-HT6 receptor or 5-hydroxytryptamine 6 receptor or serotonin 6 receptor or 5-HT7 receptor or 5-hydroxytryptamine-7 receptor or serotonin 7 receptor).mp

and

[Pain]

exp neuropathic pain/ or exp pain receptor/ or exp pain/ or exp complex regional pain syndrome/ or exp complex regional pain syndrome type II/ or exp deafferentation pain/ or exp radicular pain/ or exp experimental neuropathic pain/ or exp intractable pain/ or exp chronic pain/ or exp complex regional pain syndrome type I/ or exp discogenic pain/ or exp experimental pain/ or exp nociceptive pain/ or exp low back pain/ or exp radiculopathy/ or exp diabetic neuropathy/ or exp experimental diabetic neuropathy/ or exp failed back surgery syndrome/ or exp nociception/ or exp gate control theory/ or exp hyperalgesia/ or exp nociceptive stimulation/ or exp small fiber neuropathy/ or exp sciatic neuropathy/ or exp sciatic nerve injury/ or exp sciatica/ or exp peripheral nerve injury/

or (ache or acute pain or burning pain or crushing pain or deep pain or lightning pain or migratory pain or pain or radiating pain or splitting pain or treatment related pain or mechanonociceptor or nociceptive neuron or nociceptive receptor or nociceptor or nocireceptor or pain receptor or nociception or nociperception or pain perception or pain sensation or pain sense or pain sensitivity or small fiber neuropathy or small fibre neuropathy or small nerve fiber neuropathy or ischias or ischiatic pain or lesion of sciatic nerve or sciatic nerve disease or sciatic nerve injury or sciatic nerve lesion or sciatic nerve neuralgia-neuritis or sciatic nerve palsy or sciatic neuritis or sciatic neuropathy or sciatic pain or sciatica or chronic intractable pain or chronic pain or widespread chronic pain or atypical neuralgia or iliohypogastric nerve neuralgia or ilioinguinal neuralgia or nerve pain or neuralgia or neurodynia or neuropathic pain or paroxysmal nerve pain or perineal neuralgia or stump neuralgia or supraorbital neuralgia or vidian neuralgia or peripheral nerve damage or peripheral nerve injury or peripheral nerve trauma or central nervous system sensitization or central sensitization or chronic low back pain or loin pain or low back ache or low back pain or low backache or lowback pain or lower back pain or lumbago or lumbal pain or lumbal syndrome or lumbalgia or lumbar pain or lumbar spine syndrome or lumbar syndrome or lumbodynia or lumbosacral pain or lumbosacral root syndrome or lumbosacroiliac strain or mechanical low back pain or postural low back pain or recurrent low back pain or failed back surgery syndrome or failed back surgery or failed back syndrome or FBSS or post-laminectomy syndrome or postlaminectomy syndrome or complex regional pain syndrome or crps or algodystrophia or algodystrophic syndrome or algodystrophies or algodystrophy or algoneurodystrophy or cervical sympathetic dystrophy or complex regional pain syndrome 1 or complex regional pain syndrome type 1 or complex regional pain syndrome type I or CRPS 1 or CRPS I or CRPS type 1 or crps type i or CRPS-I or neuralgic shoulder amyotrophy or posttraumatic dystrophy or post-traumatic dystrophy or posttraumatic osteopenia or posttraumatic osteoporosis or reflex sympathetic dystrophy or reflex sympathetic dystrophy syndrome or rsd or rsds or shoulder-hand syndrome or shoulder-hand syndromes or Sudeck atrophy or Sudeck disease or Sudeck dystrophy or Sudeck Leriche syndrome or Sudeck syndrome or Sudeck's atrophy or sudeks atrophy or sudek's atrophy or Suedeck dystrophy or Suedeck reflex dystrophy or sympathetic reflex dystrophia or sympathetic reflex dystrophy or causalgia or causalgia syndrome or complex regional pain syndrome type ii or CRPS 2 or CRPS II or CRPS type 2 or crps type ii or CRPS-II or deafferentation pain or asymmetric diabetic proximal motor neuropathy or diabetes neuropathy or diabetic amyotrophy or diabetic asymmetric polyneuropathy or diabetic autonomic neuropathy or diabetic mononeuropathy or diabetic mononeuropathy simplex or diabetic neuralgia or diabetic neuritis or diabetic neuropathy or diabetic peripheral neuropathy or diabetic polyneuritis or diabetic polyneuropathy or painful diabetic neuropathy or symmetric diabetic proximal motor neuropathy or intractable pain or refractory pain or cervical radiculopathy or nerve root avulsion or nerve root compression or Nerve root disease or nerve root disorder or nerve root inflammation or neuroradiculitis or polyneuroradiculitis or Polyradiculopathy or radiculalgia or radicular neuralgia or Radicular neuropathy or radicular pain or radiculitis or radiculopathy or spinal radiculitis or Discogenic pain or allodynia or Cold allodynia or Cold hyperalgesia or Heat allodynia or Heat hyperalgesia or hyperalgesia or hyperalgia or hyperpathia or mechanical allodynia or mechanical hyperalgesia or mechanical hyperalgia or mechanical hyperpathia or tactile allodynia or thermal allodynia or thermal hyperalgesia or thermal hyperalgia or thermal hyperpathia or experimental neuropathic pain or Experimentally induced

neuropathic pain or Experimental pain or Experimentally induced pain or nociceptive pain or somatic pain or tissue pain or Experimental diabetic neuropathy or Experimentally induced diabetic neuropathy or streptozocin-induced diabetic neuropathy or streptozotocin-induced diabetic peripheral neuropathy or STZ-diabetic neuropathy or STZ-induced diabetic neuropathy or STZ-induced diabetic peripheral neuropathy or spared nerve injury or partial sciatic nerve injury or chronic constriction injury or spinal cord injury or spinal nerve ligation or spinal hemisection).mp

And

[Area]
exp spinal cord/ or exp cervical spinal cord/ or exp conus medullaris/ or exp lumbar spinal cord/ or exp sacral spinal cord/ or exp spinal cord dorsal horn/ or exp substantia gelatinosa/ or exp thoracic spinal cord/ or exp interneuron/ or exp periaqueductal gray matter/ or exp raphe magnus nucleus/ or exp somatosensory cortex/ or exp primary somatosensory cortex/ or exp secondary somatosensory cortex/ or exp rostral ventromedial medulla/ or exp spinothalamic tract/ or exp spinal ganglion/

or (cervical cord or cervical medulla or coccygeal cord or conus medullaris or conus terminalis or cornu laterale or lateral horn or lumbar cord or medulla spinalis or medullary cone or myelon or pars cervicalis medullae spinalis or pars lumbalis medullae spinalis or pars thoracica medullae spinalis or sacral cord or sacral spine or spinal cord or spinal marrow or spinal medulla or thoracic cord or clarke columns or clarke nucleus or clarkes columns or clarke's columns or clarkes nucleus or clarke's nucleus or columna posterior substantiae griseae or cornu posterius substantiae griseae or dorsal horn or marginal nucleus or nucleus dorsali or nucleus dorsalis or posterior horn or posterior horn of spinal cord or posterior spinal horn or spinal cord dorsal horn or spinal cord posterior horn or spinal dorsal column or spinal dorsal horn or spinal posterior column or zona spongiosa or lamina 2 or lamina ii or rolando substance or rolando substantia gelatinosa or substantia gelatinosa or substantia gelatinosa medullae spinalis or substantia gelatinosa of rolando or substantia gelatinosa rolandi or intercalary neuron or intercalated nerve cell or intercalated neuron or interneuron or relay nerve cell or relay neuron or central gray substance of midbrain or central periaqueductal gray or griseum centrale or griseum centrale mesencephali or griseum centrale mesencephalus or mesencephalic central gray or midbrain central gray or periaqueductal gray or periaqueductal gray matter or substantia grisea centralis or substantia grisea centralis mesencephali or nucleus raphe magnus or nucleus raphe ponti or nucleus raphe pontis or pontine raphe nucleus or raphe magnus or Raphe Magnus Nucleus or raphe ponti or raphe pontis or Raphe Pontis Nucleus or anterior parietal cortex or area S1 or area S2 or barrel cortex or first somatosensory area or first somatosensory cortex or gyrus postcentralis or neurosensory field or postcentral gyrus or primary somatic sensory area or primary somatosensory area or primary somatosensory cortex or S1 area or second somatic sensory area or second somatosensory area or second somatosensory cortex or secondary sensory cortex or secondary somatosensory area or secondary somatosensory cortex or si cortex or somate sensory cortex or somatic sensory cortex or somatosensory area or somatosensory area S1 or somatosensory area S2 or somatosensory cortex or somatosensory S1 area or spinothalamic tract or spino-thalamic tract or tractus spinothalamicus or dorsal root ganglion or dorsal root spinal ganglion or ganglion spinale or spinal dorsal root ganglion or spinal ganglion or spine ganglion or dorsal horn cell or dorsal horn neuron or posterior horn cell or posterior horn neuron or rostral ventromedial medulla).mp

And

[Neuromodulation]
exp electrostimulation/ or exp spinal cord stimulation/ or exp spinal cord stimulator/ or exp implanted spinal cord stimulator/ or exp spinal ganglion stimulator/ or exp implanted spinal ganglion stimulator/ or exp transcutaneous electrical nerve stimulation/ or exp nerve stimulation/ or exp pulsed

radiofrequency treatment/ or exp brain depth stimulation/ or exp repetitive transcranial magnetic stimulation/ or exp transcranial magnetic stimulation/ or exp transcranial direct current stimulation/ or exp transcranial electrical stimulation/ or exp magnetic stimulation/

or (Dorsal column stimulation or SCS or Dorsal root ganglion stimulation or PENS or PRF or DBS or rTMS or TMS or brain stimulation or motor cortex stimulation or electromagnetic stimulation or cortex stimulation or electric field stimulation or electric stimulation or electrical stimulation or electrostimulation or electrostimulus or galvanostimulation or spinal cord stimulation or spinal stimulation or analgesic cutaneous electrostimulation or electroanalgesia or percutaneous electric nerve stimulation or percutaneous electrical nerve stimulation or percutaneous electrical neuromodulation or percutaneous electrical or percutaneous neuromodulation therapy or tens or transcutaneous electric nerve stimulation or transcutaneous electric stimulation or transcutaneous electrical nerve stimulation or transcutaneous electrical stimulation or transcutaneous electrostimulation or transcutaneous nerve stimulation or transdermal electrostimulation or nerve stimulus or neurostimulation or peripheral nerve stimulation or nerve stimulation or pulsed radio frequency treatment or pulsed radiofrequency treatment or brain depth stimulation or brain excitation or brain stimulation or brain stimulus or deep brain stimulation or electrical brain stimulation or electrical stimulation of the brain or repetitive transcranial magnetic stimulation or transcranial magnetic stimulation or paired pulse transcranial magnetic stimulation or repetitive transcranial magnetic stimulation or single pulse transcranial magnetic stimulation or anodal stimulation tdcs or cathodal stimulation tdcs or repetitive transcranial electrical stimulation or tDCS or transcranial alternating current stimulation or transcranial direct current stimulation or transcranial electrical stimulation or transcranial random noise stimulation).mp

EMBASE (ovid): searched 15-04-2020

[Serotonin]

exp serotonin 5A receptor/ or exp serotonin 4 receptor/ or exp serotonin 2A receptor/ or exp serotonin receptor/ or exp serotonin 2B receptor/ or exp serotonin 2 receptor/ or exp serotonin/ or exp serotonin 2C receptor/ or exp serotonin 1A receptor/ or exp serotonin 7 receptor/ or exp serotonin 3 receptor/ or exp serotonin 1 receptor/ or exp serotonin 1B receptor/ or exp serotonin 1D receptor/ or exp serotonin 6 receptor/ or exp serotonin 5 receptor/ or

(3-2-aminoethyl-1h-indol-5-ol or 5 hydroxytryptamine or 5-ht or 5-hydroxytryptamine or enteramine or hippophaine or hydroxytryptamine or serotonin or 3 2 aminoethyl 5 hydroxyindole or 3 2 aminoethyl 5 indolol or 3 beta aminoethyl 5 hydroxyindole or 5 hydroxy 3 beta aminoethyl indole or 5 hydroxy tryptamine or 5 hydroxytryptamin or alpha 5 hydroxytryptamine or d,s substance or ds substance or hydroxy tryptamine or serotin or serotonine or thrombocytin or thrombotonin or 5-hydroxytryptamine receptor or serotonin receptor or tryptamine receptor or serotonergic receptor or serotoninergic receptor or 5 hydroxytryptamine 1 receptor or 5 ht1 receptor or 5-ht1 receptor or 5-ht1 serotonin receptor or serotonin 5 ht1 receptor or serotonin 5-ht1 receptor or serotonin receptor s1 or serotonin s1 receptor or serotoninergic s1 receptor or 5 ht1a receptor or 5 hydroxytryptamine 1a receptor or 5-ht1a receptor or 5-ht1a serotonin receptor or 5-ht1a receptor serotonin or 5-hydroxytryptamine 1a receptor or serotonin 1a receptor or 5 ht1b receptor or 5 ht1dbeta receptor or 5 hydroxytryptamine 1b receptor or 5-ht1b receptor or 5-ht1dbeta receptor or 5-hydroxytryptamine 1b receptor or serotonin 1b receptor or serotonin 1dbeta receptor or 5 ht1d receptor or 5 hydroxytryptamine 1d receptor or 5 hydroxytryptamine1d receptor or 5-ht1d receptor or 5-ht1dalpha receptor or 5-hydroxytryptamine1d receptor or hydroxytryptamine1d or serotonin 1d alpha receptor or serotonin 1d receptor or serotonin 1dalpha receptor or 5 ht2 receptor or 5-ht-2

receptor or 5-ht2 serotonin receptors or 5 hydroxytryptamine 2 receptor or serotonin 2 receptors or serotonin s2 receptors or serotonin-2 receptors or serotoninergic s2 receptor or 5-ht2a receptor or 5 hydroxytryptamine 2a receptor or serotonin 2a receptor or 5-ht2b receptor or 5 hydroxytryptamine 2b receptor or 5-ht2b receptor or 5-hydroxytryptamine 2b receptor or serotonin 2b receptor or 5 hydroxytryptamine 2c receptor or 5 hydroxytryptamine type 2c receptor or 5-ht2c receptor or 5-hydroxytryptamine type 2c receptor or serotonin 2c receptor or 5ht3a receptor or 5ht3b receptor or 5 hydroxytryptamine 3 receptor or 5 hydroxytryptamine 3a receptor or 5 hydroxytryptamine 3b receptor or 5-ht3 receptor or 5-hydroxytryptamine-3 receptor or serotonin 3 receptor or serotonin receptor s3 or serotonin s3 receptor or 5 hydroxytryptamine 4 receptor or 5-ht4 receptor or 5-ht4l receptor or 5-ht4s receptor or 5-hydroxytryptamine-4 receptor or serotonin 4 receptor or serotonin s4 receptor or 5-HT5 receptor or 5-hydroxytryptamine 5 receptor or 5-hydroxytryptamine 5a receptor or 5-hydroxytryptamine 5b receptor or 5HT5 receptor or serotonin 5A receptor or serotonin receptor 5A or 5-HT5A receptor or serotonin 5B receptor or 5-HT5B receptor or 5-HT6 receptor or 5-hydroxytryptamine 6 receptor or serotonin 6 receptor or 5-HT7 receptor or 5-hydroxytryptamine-7 receptor or serotonin 7 receptor).mp

and

[Pain]
exp neuropathic pain/ or exp pain receptor/ or exp pain/ or exp complex regional pain syndrome/ or exp complex regional pain syndrome type II/ or exp deafferentation pain/ or exp radicular pain/ or exp experimental neuropathic pain/ or exp intractable pain/ or exp chronic pain/ or exp complex regional pain syndrome type I/ or exp discogenic pain/ or exp experimental pain/ or exp nociceptive pain/ or exp low back pain/ or exp radiculopathy/ or exp diabetic neuropathy/ or exp experimental diabetic neuropathy/ or exp failed back surgery syndrome/ or exp nociception/ or exp gate control theory/ or exp hyperalgesia/ or exp nociceptive stimulation/ or exp small fiber neuropathy/ or exp sciatic neuropathy/ or exp sciatic nerve injury/ or exp sciatica/ or exp peripheral nerve injury/

or (ache or acute pain or burning pain or crushing pain or deep pain or lightning pain or migratory pain or pain or radiating pain or splitting pain or treatment related pain or mechanonociceptor or nociceptive neuron or nociceptive receptor or nociceptor or nocireceptor or pain receptor or nociception or nociperception or pain perception or pain sensation or pain sense or pain sensitivity or small fiber neuropathy or small fibre neuropathy or small nerve fiber neuropathy or ischias or ischiatic pain or lesion of sciatic nerve or sciatic nerve disease or sciatic nerve injury or sciatic nerve lesion or sciatic nerve neuralgia-neuritis or sciatic nerve palsy or sciatic neuritis or sciatic neuropathy or sciatic pain or sciatica or chronic intractable pain or chronic pain or widespread chronic pain or atypical neuralgia or iliohypogastric nerve neuralgia or ilioinguinal neuralgia or nerve pain or neuralgia or neurodynia or neuropathic pain or paroxysmal nerve pain or perineal neuralgia or stump neuralgia or supraorbital neuralgia or vidian neuralgia or peripheral nerve damage or peripheral nerve injury or peripheral nerve trauma or central nervous system sensitization or central sensitization or chronic low back pain or loin pain or low back ache or low back pain or low backache or lowback pain or lower back pain or lumbago or lumbal pain or lumbal syndrome or lumbalgia or lumbar pain or lumbar spine syndrome or lumbar syndrome or lumbodynia or lumbosacral pain or lumbosacral root syndrome or lumbosacroiliac strain or mechanical low back pain or postural low back pain or recurrent low back pain or failed back surgery syndrome or failed back surgery or failed back syndrome or FBSS or post-laminectomy syndrome or postlaminectomy syndrome or complex regional pain syndrome or crps or algodystrophia or algodystrophic syndrome or algodystrophies or algodystrophy or algoneurodystrophy or cervical sympathetic dystrophy or complex regional pain syndrome 1 or complex regional pain syndrome type 1 or complex regional pain syndrome type I or CRPS 1 or CRPS I or CRPS type 1 or crps type i or CRPS-I or neuralgic shoulder amyotrophy or posttraumatic dystrophy or post-traumatic

dystrophy or posttraumatic osteopenia or posttraumatic osteoporosis or reflex sympathetic dystrophy or reflex sympathetic dystrophy syndrome or rsd or rsds or shoulder-hand syndrome or shoulder-hand syndromes or Sudeck atrophy or Sudeck disease or Sudeck dystrophy or Sudeck Leriche syndrome or Sudeck syndrome or Sudeck's atrophy or sudeks atrophy or sudek's atrophy or Suedeck dystrophy or Suedeck reflex dystrophy or sympathetic reflex dystrophia or sympathetic reflex dystrophy or causalgia or causalgia syndrome or complex regional pain syndrome type ii or CRPS 2 or CRPS II or CRPS type 2 or crps type ii or CRPS-II or deafferentation pain or asymmetric diabetic proximal motor neuropathy or diabetes neuropathy or diabetic amyotrophy or diabetic asymmetric polyneuropathy or diabetic autonomic neuropathy or diabetic mononeuropathy or diabetic mononeuropathy simplex or diabetic neuralgia or diabetic neuritis or diabetic neuropathy or diabetic peripheral neuropathy or diabetic polyneuritis or diabetic polyneuropathy or painful diabetic neuropathy or symmetric diabetic proximal motor neuropathy or intractable pain or refractory pain or cervical radiculopathy or nerve root avulsion or nerve root compression or Nerve root disease or nerve root disorder or nerve root inflammation or neuroradiculitis or polyneuroradiculitis or Polyradiculopathy or radiculalgia or radicular neuralgia or Radicular neuropathy or radicular pain or radiculitis or radiculopathy or spinal radiculitis or Discogenic pain or allodynia or Cold allodynia or Cold hyperalgesia or Heat allodynia or Heat hyperalgesia or hyperalgesia or hyperalgia or hyperpathia or mechanical allodynia or mechanical hyperalgesia or mechanical hyperalgia or mechanical hyperpathia or tactile allodynia or thermal allodynia or thermal hyperalgesia or thermal hyperalgia or thermal hyperpathia or experimental neuropathic pain or Experimentally induced neuropathic pain or Experimental pain or Experimentally induced pain or nociceptive pain or somatic pain or tissue pain or Experimental diabetic neuropathy or Experimentally induced diabetic neuropathy or streptozocin-induced diabetic neuropathy or streptozotocin-induced diabetic peripheral neuropathy or STZ-diabetic neuropathy or STZ-induced diabetic neuropathy or STZ-induced diabetic peripheral neuropathy or spared nerve injury or partial sciatic nerve injury or chronic constriction injury or spinal cord injury or spinal nerve ligation or spinal hemisection).mp

And

[Area]
exp spinal cord/ or exp cervical spinal cord/ or exp conus medullaris/ or exp lumbar spinal cord/ or exp sacral spinal cord/ or exp spinal cord dorsal horn/ or exp substantia gelatinosa/ or exp thoracic spinal cord/ or exp interneuron/ or exp periaqueductal gray matter/ or exp raphe magnus nucleus/ or exp somatosensory cortex/ or exp primary somatosensory cortex/ or exp secondary somatosensory cortex/ or exp rostral ventromedial medulla/ or exp spinothalamic tract/ or exp spinal ganglion/

or (cervical cord or cervical medulla or coccygeal cord or conus medullaris or conus terminalis or cornu laterale or lateral horn or lumbar cord or medulla spinalis or medullary cone or myelon or pars cervicalis medullae spinalis or pars lumbalis medullae spinalis or pars thoracica medullae spinalis or sacral cord or sacral spine or spinal cord or spinal marrow or spinal medulla or thoracic cord or clarke columns or clarke nucleus or clarkes columns or clarke's columns or clarkes nucleus or clarke's nucleus or columna posterior substantiae griseae or cornu posterius substantiae griseae or dorsal horn or marginal nucleus or nucleus dorsali or nucleus dorsalis or posterior horn or posterior horn of spinal cord or posterior spinal horn or spinal cord dorsal horn or spinal cord posterior horn or spinal dorsal column or spinal dorsal horn or spinal posterior column or zona spongiosa or lamina 2 or lamina ii or rolando substance or rolando substantia gelatinosa or substantia gelatinosa or substantia gelatinosa medullae spinalis or substantia gelatinosa of rolando or substantia gelatinosa rolandi or intercalary neuron or intercalated nerve cell or intercalated neuron or interneuron or relay nerve cell or relay neuron or central gray substance of midbrain or central periaqueductal gray or griseum centrale or griseum centrale mesencephali or griseum centrale mesencephalus or mesencephalic central gray or midbrain central gray or periaqueductal gray or periaqueductal gray matter or substantia grisea centralis or substantia grisea centralis mesencephali

or nucleus raphe magnus or nucleus raphe ponti or nucleus raphe pontis or pontine raphe nucleus or raphe magnus or Raphe Magnus Nucleus or raphe ponti or raphe pontis or Raphe Pontis Nucleus or anterior parietal cortex or area S1 or area S2 or barrel cortex or first somatosensory area or first somatosensory cortex or gyrus postcentralis or neurosensory field or postcentral gyrus or primary somatic sensory area or primary somatosensory area or primary somatosensory cortex or S1 area or second somatic sensory area or second somatosensory area or second somatosensory cortex or secondary sensory cortex or secondary somatosensory area or secondary somatosensory cortex or si cortex or somate sensory cortex or somatic sensory cortex or somatosensory area or somatosensory area S1 or somatosensory area S2 or somatosensory cortex or somatosensory S1 area or spinothalamic tract or spino-thalamic tract or tractus spinothalamicus or dorsal root ganglion or dorsal root spinal ganglion or ganglion spinale or spinal dorsal root ganglion or spinal ganglion or spine ganglion or dorsal horn cell or dorsal horn neuron or posterior horn cell or posterior horn neuron or rostral ventromedial medulla).mp

And

[Neuromodulation]
exp electrostimulation/ or exp spinal cord stimulation/ or exp spinal cord stimulator/ or exp implanted spinal cord stimulator/ or exp spinal ganglion stimulator/ or exp implanted spinal ganglion stimulator/ or exp transcutaneous electrical nerve stimulation/ or exp nerve stimulation/ or exp pulsed radiofrequency treatment/ or exp brain depth stimulation/ or exp repetitive transcranial magnetic stimulation/ or exp transcranial magnetic stimulation/ or exp transcranial direct current stimulation/ or exp transcranial electrical stimulation/ or exp magnetic stimulation/

or (Dorsal column stimulation or SCS or Dorsal root ganglion stimulation or PENS or PRF or DBS or rTMS or TMS or brain stimulation or motor cortex stimulation or electromagnetic stimulation or cortex stimulation or electric field stimulation or electric stimulation or electrical stimulation or electrostimulation or electrostimulus or galvanostimulation or spinal cord stimulation or spinal stimulation or analgesic cutaneous electrostimulation or electroanalgesia or percutaneous electric nerve stimulation or percutaneous electrical nerve stimulation or percutaneous electrical neuromodulation or percutaneous electrical or percutaneous neuromodulation therapy or tens or transcutaneous electric nerve stimulation or transcutaneous electric stimulation or transcutaneous electrical nerve stimulation or transcutaneous electrical stimulation or transcutaneous electrostimulation or transcutaneous nerve stimulation or transdermal electrostimulation or nerve stimulus or neurostimulation or peripheral nerve stimulation or nerve stimulation or pulsed radio frequency treatment or pulsed radiofrequency treatment or brain depth stimulation or brain excitation or brain stimulation or brain stimulus or deep brain stimulation or electrical brain stimulation or electrical stimulation of the brain or repetitive transcranial magnetic stimulation or transcranial magnetic stimulation or paired pulse transcranial magnetic stimulation or repetitive transcranial magnetic stimulation or single pulse transcranial magnetic stimulation or anodal stimulation tdcs or cathodal stimulation tdcs or repetitive transcranial electrical stimulation or tDCS or transcranial alternating current stimulation or transcranial direct current stimulation or transcranial electrical stimulation or transcranial random noise stimulation).mp
