# Supplementary material for: A systematic review on descending serotonergic projections and modulation of spinal nociception in chronic neuropathic pain and after spinal cord stimulation
Source: Mol Pain. 2021 Oct 18;17:17448069211043965. doi: 10.1177/17448069211043965 (PMC8527581; doi:10.1177/17448069211043965)
Supplement: sj-pdf-2-mpx-10.1177_17448069211043965 - Supplemental material for A systematic review on descending serotonergic projections and modulation of spinal nociception in chronic neuropathic pain and after spinal cord stimulation [file sj-pdf-2-mpx-10.1177_17448069211043965.pdf]

Appendix2: Inclusion and exclusion criteria for study selection

Inclusion criteria:

- Articles must be in the English language
- Articles must be original articles (reviews and book chapters are screened for the inclusion of additional original articles)
- Studies must utilize rodents
- Studies must utilize adult animals
- Articles must include serotonin (5-HT), serotonin receptors or pharmaceuticals targeting the serotonergic system
- Studies must focus on healthy nociception or peripheral neuropathic pain models
- Treatment, if given, must target the descending serotonergic system or be spinal cord stimulation.

Exclusion criteria:

- Article is not in the English language
- Article is in any other form than an original article, review or book chapter
- Human studies
- Studies using any other kind of animal than rodents
- Developmental studies
- Articles not focused on healthy nociception of chronic neuropathic pain
- Studies using a disease model other than peripheral neuropathic pain models
- Studies using the following pain models[1,2]:
    - Inflammatory pain models, including the formalin model
    - Cancer pain models
    - Arthritic (joint) pain models
    - Muscle pain models
    - Postoperative pain models
    - Visceral pain models
    - Headache and migraine models
    - Viral neuropathy models
    - Drug- or treatment-induced pain models
    - Functional pain syndrome models
    - Stress-induced hyperalgesia models
    - Central pain models:
        - Spinal cord injury models
        - Traumatic brain injury models
        - Excitotoxicity models for stroke injury

[1] Jaggi, A.S., Jain, V. and Singh, N. (2011), Animal models of neuropathic pain. Fundamental & Clinical Pharmacology, 25: 1-28. https://doi.org/10.1111/j.1472-8206.2009.00801.x

[2] Gregory NS, Harris AL, Robinson CR, Dougherty PM, Fuchs PN, Sluka KA. An overview of animal models of pain: disease models and outcome measures. *J Pain*. 2013;14(11):1255-1269. doi:10.1016/j.jpain.2013.06.008
