# Supplementary figures and images for: A systematic review on descending serotonergic projections and modulation of spinal nociception in chronic neuropathic pain and after spinal cord stimulation
Source: Mol Pain. 2021 Oct 18;17:17448069211043965. doi: 10.1177/17448069211043965 (PMC8527581; doi:10.1177/17448069211043965)

### Appendix 3: Flowchart of study selection

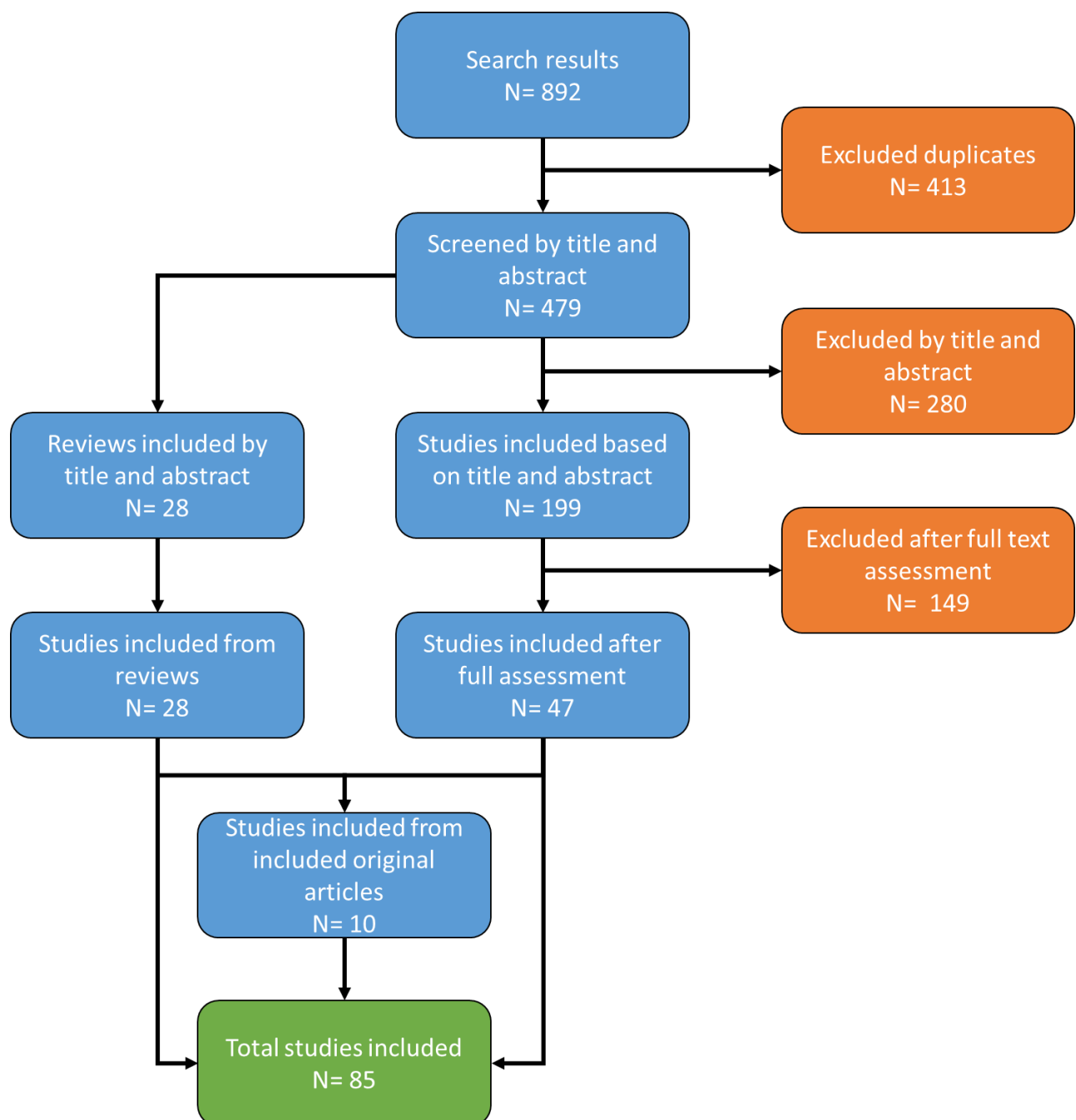

Supplement: sj-pdf-3-mpx-10.1177_17448069211043965 - Supplemental material for A systematic review on descending serotonergic projections and modulation of spinal nociception in chronic neuropathic pain and after spinal cord stimulation [file sj-pdf-3-mpx-10.1177_17448069211043965.pdf]
