# Supplementary material for: A systematic review on descending serotonergic projections and modulation of spinal nociception in chronic neuropathic pain and after spinal cord stimulation
Source: Mol Pain. 2021 Oct 18;17:17448069211043965. doi: 10.1177/17448069211043965 (PMC8527581; doi:10.1177/17448069211043965)
Supplement: sj-pdf-4-mpx-10.1177_17448069211043965 - Supplemental material for A systematic review on descending serotonergic projections and modulation of spinal nociception in chronic neuropathic pain and after spinal cord stimulation [file sj-pdf-4-mpx-10.1177_17448069211043965.pdf]

Appendix 4: Study characteristics

First author	Species	Sex	Pain model	Treatment	Assessment
Advokat et al. (9)	Rats	Male	n/a	5-HT → i.t.	Tail flick test
Aira et al. (24)	Rats	Male	L5-SNL	8-OH-DPAT (5-HT1a agonist), WAY100135 (5-HT1a antagonist), CP94253 (5-HT1b agonist), SB224289 (5-HT1b antagonist), TCB-2 5-HT2a agonist), 4F 4PP (5-HT2a antagonist), BW723C86 (5-HT2b agonist), SB204741 (5-HT2b antagonist), WAY161503 (5-HT2c agonist), RS102221 (5-HT2c antagonist), SR57227 (5-HT3 agonist), Y-25130 (5-HT3 antagonist), RS67333 (5-HT4 agonist), RS39604 (5-HT4 antagonist) → spinal superfusion	Von Frey test<br>Electrophysiology
Aira et al. (86)	Rats	Male	L5 SNL	TCB-2 (5-HT2a agonist) → spinal superfusion<br>M100907 (5-HT2a antagonist) → i.p.	Electrophysiology<br>Von Frey test<br>Hot-plate test
Aira et al. (88)	Rats	Male	L5 SNL	4F 4PP (5-HT2a antagonists), SB204741 (5-HT2b antagonist) → spinal superfusion	Von Frey test<br>Electrophysiology<br>Immunohistochemistry
Alhaider et al. (30)	Mice<br>Rats	Male	n/a	2-methyl serotonin (5-HT3 agonist, zacopride (5-HT3 antagonist), ICS 205-930 (5-HT3 antagonist) → i.t.<br>2-methyl serotonin (5-HT3 agonist), zacopride (5-HT3 antagonist) → spinal iontoporetic application	Tail flick test (mice)<br>Electrophysiology (rats)

Ali et al. (26)	Rats	Male	n/a	5-HT, 5-CT, TFMPP (5-HT1b agonist), 8-OH-DPAT (5-HT1a agonist) → i.t.	Tail flick test<br>Electrophysiology
Amaya-Castellanos et al. (93)	Rats	Female	L5/6-SNL	SB-269970 (5-HT7 antagonist) → i.t., i.p.	Von Frey test<br>Western blot
Ardid et al. (38)	Rats	Male	CCI<br>PDPN	WAY 100,635 (5-HT1a antagonist), Clomipramine (TCA) → i.v.	Paw pressure test
Avila-Rojas et al. (74)	Rats	Female	L5/6-SNL	5-HT, 5-CT, GR-127935 (5-HT1b/d antagonist), methiothepin (nonselective 5-HT antagonist), SB-699551 (5-HT5a antagonist), WAY-100635 (5-HT1a antagonist)	Von Frey test<br>Western blot
Bee et al. (42)	Rats	Male	Intact<br>L5/6-SNL	Ondansetron (5-HT3 antagonist), 2-methyl 5-HT (5-HT3 agonist) → spinal application	Von Frey test<br>Electrophysiology
Berge et al. (16)	Rats	Male	n/a	5-MeODMT (nonselective 5-HT agonist) → s.c.<br>5-HTP (5-HT precursor), mianserin (TCA), metergoline (nonspecific 5-HT antagonist) → i.p.	Tail flick test
Bonnefont et al. (39)	Rats	Male	n/a	5-HT, WAY-100365 (5-HT1a antagonist) → i.t.	Paw pressure test
Brenchat et al. (49)	Mice	Male	PSNL	AS-19 (5-HT7 agonist), SB-258719 (5-HT7 antagonist), E-57431 (5-HT7 agonist) → s.c. or i.p.	Von Frey test<br>Plantar test (radiant heat)<br>Immunohistochemistry
Brenchat et al. (58)	Rats	Male	SNI	5-57431 (5-HT7 agonist) → i.t. and i.p.	Von Frey test
Cai et al. (37)	Mice	Male	n/a	Optogenetic activation of TPH2-channel rhodopsin in the RVM	Von Frey test<br>Plantar test (radiant heat)

Chang et al. (89)	Mice	Male	L4-SNL	Ondansetron (5-HT3 antagonist) → i.t. and i.p.	Von Frey test<br>Hot box test
Colpaert et al. (18)	Rats	Male	CCI	F13640 (5-HT1a agonist) → s.c. and i.p.	Automated Von Frey test
Conte et al. (45)	Rats	Male	n/a	n/a	5-HT3a immunohistochemistry
Cragg et al. (83)	Rats	Male	C7/8 DRI	Spinal 5-HT depletion by 5.7-DHT	Adhesive removal test<br>Acetone test<br>Immunohistochemistry
Daval et al. (60)	Rats	Male	Neonatal capsaicin<br>C4-6 rhizotomy	n/a	Radioligand binding assay for 5-HT and 5-HT1a
Dogrul et al. (90)	Rats	Male	L5/6 SNL	Ondansetron (5-HT3 antagonist), SB-269970 (5-HT7 antagonist)	Paw flick test (radiant heat)<br>Von Frey test
Doly et al. (36)	Rats<br>Mice	Male	n/a	n/a	5-HT5a immunohistochemistry<br>5-HT5a electron microscopy
Doly et al. (56)	Rats<br>Mice	Male	n/a	n/a	5-HT2a immunohistochemistry<br>5-HT2a electron microscopy
Doly et al. (59)	Rats	Male	n/a	n/a	5-HT7 immunohistochemistry<br>5-HT7 electron microscopy
Fasmer et al. (12)	Mice	Male	n/a	Spinal 5-HT depletion by 5.6-DHT	Tail flick test
Gautier et al. (68)	Rats	Male	CCI	Inhibition of TPH2 expression (i.e. 5-HT depletion) in RVM by shRNA interference	Von Frey test<br>Paw pressure test<br>Hargreaves test
Gjerstad et al. (21)	Rats	Female	n/a	8-OH-DPAT (5-HT1a agonist), WAY100635 (5-HT1a antagonist) → spinal application	Electrophysiology
Gjerstad et al. (25)	Rats	Female	n/a	CP-93,129 (5-HT1b agonist), cyanopindolol (5-HT1a/b antagonist), WAY100635 (5-HT1a antagonist) → spinal application	Electrophysiology
Glaum et al. (31)	Rats	Male	n/a	5-HT, 2-methyl 5-HT (5-HT3 agonist), ICS 205-930 (5-HT3	Tail flick test<br>Hot plate test

				antagonist), MDL 72222 (5-HT3 antagonist) → i.t.	
Guo et al. (43)	Rats	Male	L5-SNL	Intra-RVM TPH2 shRNA interference (i.e. 5-HT depletion)<br>SR-57227 (5-HT3 agonist) → i.t.	Von Frey test<br>Paw withdrawal test to radiant heat
Honda et al. (79)	Mice	Male	PSNL	5-HT depletion by i.c.v. 5,7-DHT injection<br>Fluvoxamine (SSRI), Amitriptyline (TCA), WAY100635 (5-HT1a antagonist), ketanserin (5-HT2a/c antagonist), Granisetron (5-HT3 antagonist) → i.c.v., s.c., i.p. or i.t.	Von Frey test<br>Paw pressure test
Hoshino et al. (72)	Rats	Male	L5-SNL	Duloxetine (SNRI), amitriptyline (TCA) → i.p.	Paw pressure test<br>Microdialyis<br>HPLC
Huang et al. (46)	Mice	Not specified	n/a	n/a	5-HT3a in situ hybridization histochemistry combinded with immunohistochemical labeling
Jones et al. (55)	Rats	Male	n/a	n/a	5-HT immunohistochemistry and Anterograde tracer in NRM
Kawamata et al. (57)	Rats	Male	n/a	1-phenylbiguanide (5-HT3 agonist), 3-tropanyl-indole-3-carboxylate methiodide (5-HT3 antagonist) → microdialysis perfusion	Microdialysis + HPLC
Kidd et al. (47)	Rats	Male	Neonatal capsaicin<br>C4-T2 dorsal rhizotomy	5-HT lesioning by 5.7-DHT in DRN	5-HT1a and 5-HT3 radioligand binding assay
Kim et al. (80)	Mice	Male	CCI	Intra-RVM TPH2 depletion by RNA interference (i.e. 5-HT depletion)	Von Frey test<br>Calcium imaging (ex vivo)<br>Whole-cell patch-clamp recordings

				SR57227 (5-HT3 agonist), Y25130 (5-HT3 antagonist) → slice application	
Kuraishi et al. (8)	Rats	Male	n/a	5-HT → i.t.	Tail flick test<br>Hot plate test<br>Tail pinch test
Laporte et al. (27)	Rats	Male	C4-T2 dorsal rhizotomy	5-HT depletion by 5,7-DHT in cisterna magna	HPLC<br>Quantitative autoradiography of 5-HT1a, 5-HT1b, 5-HT3
Leong et al. (65)	Rats	Male	L5-SNL	Intra-RVM 5-HT depletion by 5,7-DHT	Von Frey test<br>Immunohistochemistry
Lin et al. (19)	Rats	Male	n/a	8-OH-DPAT (5-HT1a agonist), CGS-12066B (5-HT1b agonist), s-(-)-propranolol (5-HT1a antagonist) → i.t. and iontophoretic application	Hargreaves test<br>Electrophysiology
Liu et al. (10)	Rats	Male	n/a	5-HT, WAY 100635 (5-HT1a antagonist), GR 55562 (5-HT1b antagonist), ketanserin (5-HT2a antagonist), RS 102221 (5-HT2c antagonist), MDL 72222 (5-HT3 antagonist), GR 113808 (5-HT4 antagonist), 8-OH-DPAT (5-HT1a agonist), CGS 12066 (5-HT1b agonist), α-methyl-5-HT (5-HT2a agonist), MK 212 (5-HT2c agonist), mCPBG (5-HT3 agonist), BTZT (5-HT4 agonist) → spinal application	Electrophysiology
Liu et al. (35)	Rats	Male	L5-SNL	5-HT, WAY 100635 (5-HT1a antagonist), GR 55562 (5-HT1b antagonist), ketanserin (5-HT2a antagonist), RS 102221 (5-HT2c antagonist), MDL 72222 (5-HT3 antagonist), GR 113808 (5-HT4 antagonist), 8-OH-DPAT (5-	Von Frey test<br>Electrophysiology<br>HPLC

				HT1a agonist), CGS 12066 (5-HT1b agonist), α-methyl-5-HT (5-HT2a agonist), MK 212 (5-HT2c agonist), mCPBG (5-HT3 agonist), BTZT (5-HT4 agonist) → spinal application	
Lopez-Alvarez et al. (87)	Rats	Female	SNTR	Ketanserin (5-HT2a antagonist) → i.p.	Automated Von Frey test<br>Plantar test (radiant heat)<br>Immunohistochemistry
Lu et al. (11)	Rats	Not specified	n/a	5-HT → superfusion	Tight-seal whole-cell patch-clamp recording
Maxwell et al. (48)	Rats	Male	n/a	n/a	5-HT3a immunohistochemistry<br>5-HT3a electron microscopy
Meller et al. (17)	Rats	Male	Intact	5-HT → i.v.	Tail flick test
Monroe et al. (28)	Rats	Male	n/a	[^{3}H]5-HT → incubation<br>Methiothepin (nonspecific 5-HT antagonist), spiperone (5-HT2 antagonist), ketanserin (5-HT2 antagonist), quipazine (5-HT1b antagonist), fluoxetine (SSRI), LSD, desmethylimipramine (TCA) → superfusion	Standard [^{3}H]5-HT release assay<br>Transmitter accumulation assay<br>Ligand binding assay
Morgado et al. (69)	Rats	Male	Diabetes	n/a	Randall-Selitto test<br>5-HT ELISA<br>Immunohistochemistry
Nadeson et al. (20)	Rats	Male	n/a	1-(3-chlorophenyl)-piperazine dihydrochloride (nonselective 5-HT1 agonist), 8-OH-DPAT (5-HT1a agonist), [4-[3-(benzotriazol-1-yl)propyl]-1-(2-methoxyphenyl)-piperazine] (5-HT1a antagonist) → i.t.	Electric current threshold test<br>Tail flick test
Nitanda et al. (29)	Rats	Male	CCI	NAN-190 (5-HT1a antagonist), (-)-pindolol (5-HT1a/b antagonist), tropisetron (5-	Paw pressure test<br>HPLC

				HT3/4 antagonist), methysergide (nonselective 5-HT2a antagonist), sarpogrelate (5-HT2a antagonist), ketanserin (5-HT2a antagonist) → i.p. and topical and s.c.	5-HT2a receptor ligand binding assay
Obata et al. (76)	Rats	Male	L5/6-SNL	MK212 (5-HT2c agonist), mCPP (5-HT2c agonist), TFMPP (5-HT2c agonist), BW723C86 (5-HT2b agonist), ketanserin (5-HT2a antagonist), RS-102221 (5-HT2c antagonist), DOI (5-HT2c agonist), α-methyl-5-HT (5-HT2c agonist) → i.t.	Von Frey test
Obata et al. (77)	Rats	Male	L5/L6-SNL	α-methyl-5-HT (5-HT2 agonist) → i.t.	Von Frey test
Okazaki et al. (81)	Rats	Male	L5-SNL	Ketanserin (5-HT2a antagonist), m-CPBG (5-HT3 agonist), MDL72,222 (5-HT3 antagonist) → i.t.	Von Frey test
Patel et al. (44)	Rats	Male	L5/6-SNL	Ondansetron (5-HT3 antagonist), ketanserin (5-HT2a antagonist) → spinal application	Von Frey<br>Electrophysiology
Paul et al. (32)	Mice	Male	n/a	Antisense oligodeoxynucleosides against 5-HT1b and 5-HT3 (i.e. knock down of receptors) → i.t.<br>5-HT, CGS-12066A (5-HT1b agonist), 2-methyl-5-HT (5-HT3 agonist) → i.t.	Tail flick test<br>5-HT1b receptor binding assay<br>5-HT3 receptor binding assay
Peng et al. (22)	Rats	Not specified	n/a	Ondansetron (5-HT3 antagonist), zacopride (5-HT3 antagonist), 1-phenylbiguanide (5-HT3 agonist), 8-OH-DPAT (5-HT1a agonist) → intraspinal via microdialysis	Electrophysiology

Peng et al. (34)	Rats	Not specified	n/a	1-phenylbiguanide (5-HT3 agonist), ondansetron (5-HT3 antagonist) → spinal application	Electrophysiology
Pertovaara et al. (82)	Rats	Male	L5/6-SNL	Methysergide (nonselective 5-HT antagonist) → i.t.	Von Frey test<br>Paw pressure test<br>Tail flick test<br>Electrophysiology
Peters et al. (73)	Rats	Male	L5/6-SNL	Ondansetron (5-HT3 antagonist), dolasetron (5-HT3 antagonist) → i.t.	Von Frey test<br>Randal-Selitto paw pressure test<br>Paw withdrawal test to radiant heat<br>Immunohistochemistry<br>HPLC
Polgár et al. (54)	Rats	Male	n/a	n/a	5-HT immunocytochemistry and retrograde tracer from caudal ventrolateral medulla<br>Electron microscopy
Qu et al. (13)	Rats	Male	n/a	5-HT, NAN-190 (5-HT1a antagonist), CPT (5-HT2 antagonist), LY-278,584 (5-HT3 antagonist), GR 113808 (5-HT4 antagonist → intra-ventrolateral orbital cortex	Tail flick test
Rahman et al. (41)	Rats	Male	n/a	Ketanserin (5-HT2a/c antagonist), DOI (5-HT2a/c agonist) → spinal application<br>Ritanserin (5-HT2a/c antagonist) → s.c.	Electrophysiology
Rahman et al. (85)	Rats	Male	L5/6-SNL	Spinal 5-HT depletion by 5,7-DHT → i.t.	Immunohistochemistry<br>Electrophysiology<br>Von Frey test<br>Acetone test
Ramer et al. (70)	Rats	Male	Rhizotomy of C8, C7/8, C5/6-T1/2 and C4-T2	n/a	Paw pressure test<br>Acetone test<br>Plantar test (radiant heat)<br>5-HT immunohistochemistry

Saadé et al. (104)	Rats	Not specified	SNI<br>DLF lesion at C6-C7	Ketanserin (5-HT2 antagonist), methysergide (5-HT1/2 antagonist) → i.p.<br><br>DCNS (rostral) & SCS (caudal) → conventional, 70% MT, 0.2 ms pulse width, 50 Hz for 5 minutes	Von Frey test<br>Acetone test<br>Paw withdrawal test to radiant heat
Sasaki et al. (78)	Rats	Male	CCI	α-methyl-5-HT (5-HT2a/c agonist), ketanserin (5-HT2a/c antagonist) → i.t.	Hargreaves test
Satoh et al. (71)	Rats	Male	CCI	Methysergide (nonspecific 5-HT antagonist) → i.t.	Plantar test (radiant heat)<br>HPLC
Scott et al. (33)	Rats<br>Mice	Female (rats)<br>Male (mice)	n/a	Ondansetron (5-HT3 antagonist) → i.t.	Paw withdrawal test to radiant heat
Song et al. (66)	Rats	Male	PSNL	5-HT → i.t.<br><br>SCS (T11) → conventional, monopolar, 80% MT, 0.2 ms pulse width, 50 Hz for 30 minutes	Von Frey test<br>Ethyl chloride test<br>Plantar test (radiant heat)<br>5-HT ELISA<br>Immunohistochemistry
Song et al. (102)	Rats	Male	SNI	SCS (T11) → conventional, monopolar, 80% MT, 0.2 ms pulse width, 50 Hz for 30 minutes	Von Frey test<br>Electrophysiology
Song et al. (104)	Rats	Male	PSNL	Methiothepin (5-HT1/6/7 antagonist), ketanserin (5-HT2a antagonist), α-methyl-5-HT (5-HT2 agonist), m-CPBG (5-HT3 agonist), TICM (5-HT3 antagonist), SDZ-205,5578,SDZ (5-HT4 antagonist) → i.t.	Von Frey test<br>Ethyl chloride test<br>Plantar test (radiant heat)

				SCS (T11) → conventional, monopolar, 80% MT, 0.2 ms pulse width, 50 for 30 minutes	
Suzuki et al. (92)	Rats	Male	L5/6-SNL	Ondansetron (5-HT3 antagonist) → spinal application	Von Frey test<br>Electrophysiology
Tazawa et al. (103)	Rats	Male	L5-SNL	Methysergide (5-HT1/2 antagonist) → i.t.<br><br>SCS (T10-11) and (L4-5) → conventional, monopolar, 70-80% MT, 0.2 ms pulse width, 50 Hz for 3 hours	Electronic Von Frey<br>Immunohistochemistry<br>Western blot
Van Steenwinckel et al. (61)	Rats	Not specified	n/a	n/a	5-HT2a immunohistochemistry<br>5-HT2a electron microscopy<br>HPLC
Viguier et al. (50)	Rats	Male	CCI	AS-19 (5-HT7 agonist), E-55888 (5-HT7 agonist), MSD-5a (5-HT7 agonist) → s.c. and i.t.<br>SB-269970 (5-HT7 antagonist/inverse agonist)→ i.p. and i.t.	Paw pressure test<br>Von Frey test<br>Paw immersion test
Vogel et al. (67)	Mice	Female	CCI	5-HT transporter knock out	Hargreaves test<br>Von Frey test<br>Acetone test<br>HPLC
Wang et al. (40)	Mice	Male	n/a	n/a	5-HT1a immunohistochemistry<br>5-HT2a immunohistochemistry
Wang et al. (91)	Rats	Male	L5/6-SNL<br>DLF lesion (T8)	Ondansetron (5-HT3 antagonist) → i.t.	Von Frey test<br>Paw withdrawal test to radiant heat<br>Conditioned placed preference test
Wei et al. (75)	Rats	Male	L5/6-SNL	WAY-100635 (5-HT1a antagonist) → intra RVM and i.t.	Paw pressure test<br>Tail flick test

Wei et al. (84)	Rats	Male	L5-SNL	Intra-RVM TPH2 shRNA interference (i.e. 5-HT depletion)	Von Frey test<br>Immunohistochemistry<br>Western Blot<br>5-HT ELISA
Xiao et al. (14)	Rats	Not specified	n/a	5-HT, cyproheptadine (5-HT2 antagonist) → intra-thalamic nucleus submedius	Tail flick test
Xiao et al. (15)	Rats	Not specified	n/a	5-HT, p-MPPI (5-HT1a antagonist), LY-278,584 (5-HT3 antagonist) → intra-thalamic nucleus submedius	Tail flick test
You et al. (23)	Rats	Male	n/a	F 13640 (5-HT1a agonist), WAY 100635 (5-HT1a antagonist) → i.p.	Electrophysiology<br>Single motor unit EMG
Zeitz et al. (62)	Mice	Not specified	Intact<br>PSNL	5-HT3a knock out<br>5-HT → i.p.<br>mCPBG (5-HT3 agonist) → superfusion	Hot plate test<br>Tail flick test<br>Tail pinch test<br>Hargreaves tsst<br>i.p. acetic acid test<br>5-HT3 radioligand binding assay<br>Electrophysiology<br>Whole-cell clamp

5,6-DHT, 5,6-dihydroxytryptamine; 5,7-DHT, 5,7-dihydroxytryptamine; 5-CT, 5-carboxamidotryptamine; 5-HT, 5-hydroxytryptamine (i.e. serotonin); CCI, chronic constriction injury; DCNS, dorsal column nuclei stimulation; DLF, dorsolateral funiculus; DRI, dorsal root injury; DRN, dorsal raphe nuclei; ELISA, enzyme-linked immunosorbent assay; EMG, electromyography; HPLC, high-performance liquid chromatography; i.c.v., intracerebroventricular; i.p., intraperitoneal; i.t., intrathecal; i.v., intravenous; MT, motor threshold; n/a, not applicable (i.e. no pain model or no treatment described); NRM, nucleus raphe magnus; PDPN, painful diabetic polyneuropathy; PSNL, partial sciatic nerve ligation; RVM, rostral ventromedial medulla; s.c., subcutaneous; SCS, spinal cord stimulation; shRNA, short hairpin ribonucleic acid; SNI, spared nerve injury; SNL, spinal nerve ligation; SNRI, serotonin-noradrenalin reuptake inhibitor; SNTR, spinal nerve transection and repair; SSRI, selective serotonin reuptake inhibitor; TCA, tricyclic antidepressant; TPH2, tryptophan hydroxylase 2
