# Supplementary material for: A systematic review on descending serotonergic projections and modulation of spinal nociception in chronic neuropathic pain and after spinal cord stimulation
Source: Mol Pain. 2021 Oct 18;17:17448069211043965. doi: 10.1177/17448069211043965 (PMC8527581; doi:10.1177/17448069211043965)
Supplement: sj-pdf-5-mpx-10.1177_17448069211043965 - Supplemental material for A systematic review on descending serotonergic projections and modulation of spinal nociception in chronic neuropathic pain and after spinal cord stimulation [file sj-pdf-5-mpx-10.1177_17448069211043965.pdf]

Appendix 5 : Risk of bias analysis

Table 2: SYRCLE risk of bias quality assessment
Low risk of bias: Green cells marked with a check mark or n/a. N/a entails that the study design is robust for the allocated type of bias.
Risk of bias could not be determined: Orange cells marked with a question mark.
High risk of bias: Red cells marked with a cross.

[insert table RoB]
